# Early Growth Response 1 Deficiency Protects the Host against Pseudomonas aeruginosa Lung Infection

**DOI:** 10.1128/IAI.00678-19

**Published:** 2019-12-17

**Authors:** Zheng Pang, Renee Raudonis, Craig McCormick, Zhenyu Cheng

**Affiliations:** a Department of Pathology, Dalhousie University, Halifax, Nova Scotia, Canada; b Department of Microbiology & Immunology, Dalhousie University, Halifax, Nova Scotia, Canada; Georgia Institute of Technology School of Biological Sciences

**Keywords:** *Pseudomonas aeruginosa*, Egr-1, inflammation

## Abstract

Pseudomonas aeruginosa is an opportunistic pathogen that is a common cause of nosocomial infections. The molecular mechanisms governing immune responses to P. aeruginosa infection remain incompletely defined. Early growth response 1 (Egr-1) is a zinc-finger transcription factor that controls inflammatory responses. Here, we characterized the role of Egr-1 in host defense against P. aeruginosa infection in a mouse model of acute bacterial pneumonia. Egr-1 expression was rapidly and transiently induced in response to P. aeruginosa infection. Egr-1-deficient mice displayed decreased mortality, reduced levels of proinflammatory cytokines (tumor necrosis factor [TNF], interleukin-1β [IL-1β], IL-6, IL-12, and IL-17), and enhanced bacterial clearance from the lung. Egr-1 deficiency caused diminished NF-κB activation in P. aeruginosa-infected macrophages independently of IκBα phosphorylation. A physical interaction between Egr-1 and NF-κB p65 was found in P. aeruginosa-infected macrophages, suggesting that Egr-1 could be required for assembly of heterodimeric transcription factors that direct synthesis of inflammatory mediators. Interestingly, Egr-1 deficiency had no impact on neutrophil recruitment *in vivo* due to its differential effects on chemokine production, which included diminished accumulation of KC (CXCL1), MIP2 (CXCL2), and IP-10 (CXCL10) and increased accumulation of LIX (CXCL5). Importantly, Egr-1-deficient macrophages and neutrophils displayed significant increases in nitric oxide production and bacterial killing ability that correlated with enhanced bacterial clearance in Egr-1-deficient mice. Together, these findings suggest that Egr-1 plays a detrimental role in host defense against P. aeruginosa acute lung infection by promoting systemic inflammation and negatively regulating the nitric oxide production that normally assists with bacterial clearance.

## Abstract (precis)

Pseudomonas aeruginosa is an opportunistic pathogen that is a common cause of nosocomial infections. The molecular mechanisms governing immune responses to P. aeruginosa infection remain incompletely defined. Early growth response 1 (Egr-1) is a zinc-finger transcription factor that controls inflammatory responses. Here, we characterized the role of Egr-1 in host defense against P. aeruginosa infection in a mouse model of acute bacterial pneumonia.

## INTRODUCTION

Pseudomonas aeruginosa is an opportunistic pathogen that causes significant morbidity and mortality in cystic fibrosis patients and immunocompromised individuals (1). Normally, efficient clearance of pulmonary P. aeruginosa infections requires the proinflammatory cytokines and chemokines that direct immune cell recruitment to the site of infection (2). However, excessive and sustained production of proinflammatory cytokines can cause systemic inflammation, severe tissue damage, and death (3, 4). Systemic inflammation in response to P. aeruginosa infection has been shown in humans and other mammals (5–7). A tightly controlled inflammation level ensures effective host defense in response to bacterial infection and maintenance of tissue homeostasis (8). However, the molecular mechanisms controlling host immune responses to P. aeruginosa infection remain incompletely defined.

Early growth response 1 (Egr-1), also known as NGFI-A, Krox24, Tis8, Zif268, and ZENK (9), is a zinc-finger transcription factor that binds to a GC-rich consensus promoter sequence, GCG(G/T)GGGCG, and transactivates genes that regulate cell growth, migration, differentiation, and apoptosis (10–13). Egr-1 is broadly expressed in different cell types (14) and, as its name suggests, is rapidly induced by a wide range of stimuli, including growth factors, cytokines, stress, and injury (15–18). Egr-1 can function as either a transcriptional activator or a repressor (10, 19). Binding of transcriptional corepressors NGFI-A binding protein 1 (NAB1) and NAB2 to the inhibitory domain of Egr-1 causes repression of Egr-1-mediated gene transcription (20, 21). Egr-1 can also bind and modulate the activity of NF-κB and NFAT transcription factors (22, 23). Elevated Egr-1 expression has been linked to production of inflammatory mediators in pulmonary diseases (24–26). However, the role of Egr-1 in host defense against P. aeruginosa lung infection has not been elucidated.

In this study, we used a mouse model of bacterial pneumonia to examine the biological implications of the presence of Egr-1 during P. aeruginosa infection. We found that Egr-1 expression was rapidly and transiently induced by P. aeruginosa in both mouse lung tissues and macrophages. Furthermore, Egr-1 deficiency resulted in less mortality and enhanced bacterial clearance without affecting neutrophil recruitment but was associated with elevated nitric oxide (NO) levels during P. aeruginosa lung infection. The levels of proinflammatory cytokines tumor necrosis factor (TNF), interleukin-1β (IL-1β), IL-6, IL-12, and IL-17 were significantly decreased in infected Egr-1-deficient mice. Interestingly, Egr-1 deficiency had differential impacts on chemokine production *in vivo*, which resulted in impaired production of KC (CXCL1), MIP2 (CXCL2), and IP-10 (CXCL10) but upregulated production of LIX (CXCL5). *In vitro* studies revealed that Egr-1-deficient neutrophils and macrophages had enhanced intracellular bacterial killing ability, which correlated with increased nitric oxide production. Further study revealed a physical interaction between Egr-1 and NF-κB p65 in P. aeruginosa-infected macrophages. These findings suggest that Egr-1 deficiency protects the host against P. aeruginosa by reducing the risk of systemic inflammation and upregulating nitric oxide production for bacterial clearance.

## RESULTS

### Egr-1 deficiency decreases mortality and enhances bacterial clearance but has no effect on neutrophil recruitment during P. aeruginosa lung infection.

**FIG 1:**
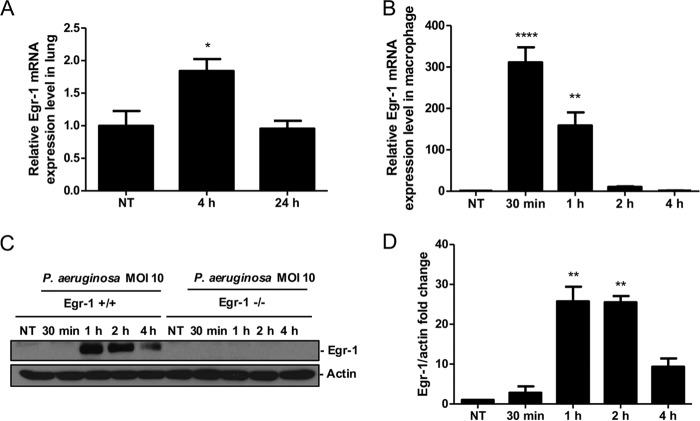
Egr-1 expression is induced in response to P. aeruginosa infection both *in vivo* and *in vitro*. Wild-type (+/+) and Egr-1-deficient (−/−) mice were intranasally infected with 1 × 10^9^ CFU/mouse of P. aeruginosa 8821 for 4 h or 24 h or with an equivalent volume of saline solution as a control (NT). The total RNA extracted from lungs was reverse transcribed to cDNA and subjected to real-time quantitative PCR for Egr-1 gene expression. The gene expression was normalized to the HPRT housekeeping control gene (A) (*n* = 3 ± SEM; *, *P* < 0.05). BMMs were infected with P. aeruginosa strain 8821 at an MOI of 10 for 30 min, 1 h, 2 h, or 4 h or left untreated (NT). Total RNA isolated from these cells was reverse transcribed to cDNA and subjected to real-time quantitative PCR for Egr-1 gene expression. The Egr-1 mRNA levels were normalized to endogenous control HPRT (B) (*n* = 3 ± SEM; **, *P* < 0.01; ****, *P* < 0.0001). Cell lysates were subjected to Western blotting for Egr-1 protein expression, and actin was used as a loading control. Blots are representative of three independent experiments (C). Densitometry analysis of Egr-1 protein levels was normalized to actin, and data are presented as fold change (D) (*n* = 3 ± SEM; **, *P* < 0.01).

**FIG 2:**
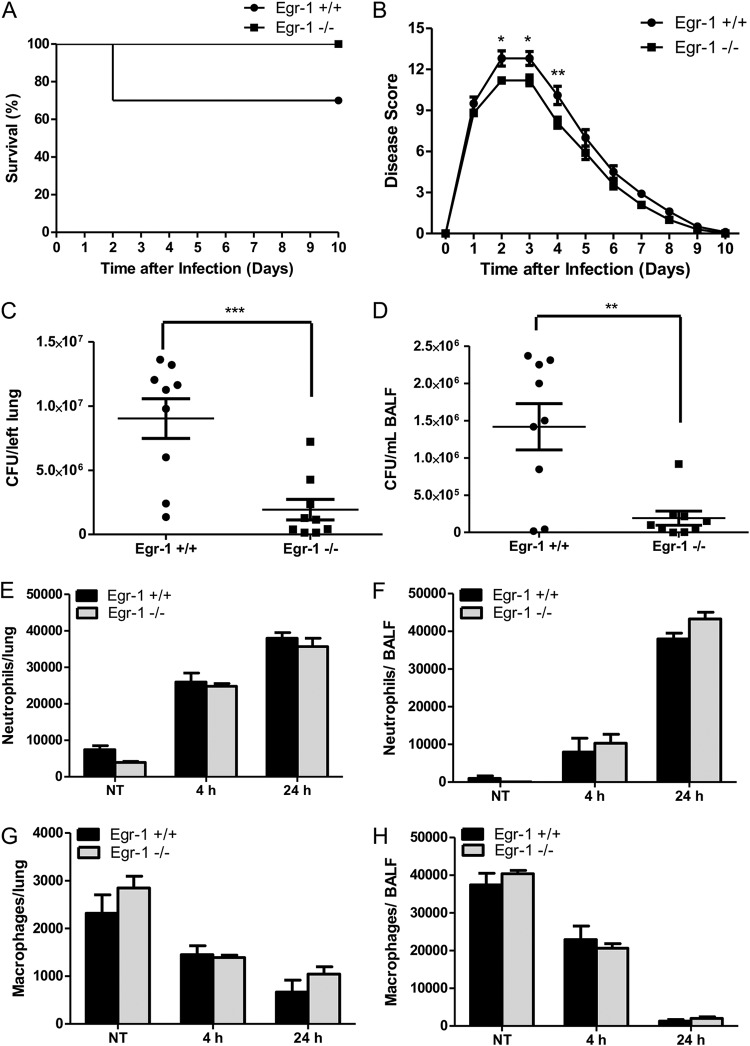
Egr-1 deficiency decreases mortality and enhances bacterial clearance but has no effect on neutrophil recruitment during P. aeruginosa infection *in vivo*. Wild-type (+/+) and Egr-1-deficient (−/−) mice were intranasally infected with 1 × 10^9^ CFU/mouse of P. aeruginosa 8821 or an equivalent volume of saline solution as a control (NT). For survival study, mice were monitored daily up to 10 days (A), and the disease scores were calculated daily (B) (*n* = 10 ± SEM; *, *P* < 0.05; **, *P* < 0.01). To determine bacterial clearance, the bacterial burden in the lung (C) and BALF (D) was assessed at 24 h (*n* = 9; **, *P* < 0.01; ***, *P* < 0.001). For immune cell recruitment studies, lung tissues and BALF were collected at 4 h or 24 h postinfection. The numbers of neutrophils (E and F) and macrophages (G and H) that infiltrated into the lung (E and G) and BALF (F and H) were determined by flow cytometry analysis at 4 h and 24 h. A total of 5 × 10^4^ cells from one lung or BALF sample was analyzed on a flow cytometer, and data are presented as cell numbers (*n* = 5).

Aberrant Egr-1 expression has been implicated in pulmonary inflammatory diseases (24–26). We first identified an increase of Egr-1 mRNA levels in lung at 4 h following P. aeruginosa 8821 infection, which suggested that Egr-1 may be involved in regulation of P. aeruginosa-induced inflammatory responses *in vivo* (Fig. 1A). Moreover, Egr-1 mRNA and protein levels were highly upregulated in macrophages in response to P. aeruginosa infection *in vitro* (Fig. 1B to D). To determine the biological implications of Egr-1 induction during P. aeruginosa lung infection, we assessed mortality and bacterial clearance using a mouse model of acute bacterial pneumonia. Wild-type and Egr-1-deficient mice were intranasally infected with P. aeruginosa 8821 and monitored for 10 days postinfection. No mortality was observed in Egr-1-deficient mice whereas 30% mortality of wild-type mice was observed by 2 days postinfection (Fig. 2A). However, the difference did not reach statistical significance using the log-rank test. Furthermore, Egr-1-deficient mice displayed significantly decreased disease scores from day 2 to day 4 of infection compared to wild-type mice (Fig. 2B). No mortality was observed after day 2 of postinfection due to recovery from infection in mice, which was found to be associated with reduced disease scores during subsequent postinfection days. These findings suggest that Egr-1 has deleterious effects on the host during P. aeruginosa lung infection. To further characterize the effect of Egr-1 on bacterial clearance during P. aeruginosa lung infection, we assessed the bacterial burden in the lungs and bronchoalveolar lavage fluid (BALF) of wild-type and Egr-1-deficient mice at 24 h postinfection by CFU counting. Egr-1-deficient mice had lower levels of bacterial burden in lungs (Fig. 2C) and BALF (Fig. 2D) than wild-type mice, suggesting that Egr-1 deficiency promotes bacterial clearance of P. aeruginosa. Neutrophils and macrophages are major phagocytic cells that are important for combating P. aeruginosa infection in the lung (27). To examine whether Egr-1 influences pulmonary infiltration of neutrophils and macrophages, we extracted the cells from P. aeruginosa-infected lungs and BALF and analyzed them by flow cytometry. We found that the number of neutrophils was increased whereas the number of macrophages was decreased in the lung and BALF cell populations of wild-type and Egr-1-deficient mice upon P. aeruginosa infection (Fig. 2E to H). However, the numbers of neutrophils and macrophages in the lung and BALF showed no significant difference between wild-type and Egr-1-deficient mice. These findings suggest that Egr-1 does not affect the recruitment of neutrophils and macrophages in response to P. aeruginosa infection and that the enhanced bacterial clearance is mediated through other mechanisms.

### Egr-1 deficiency impairs proinflammatory cytokine production but has differential effects on chemokine production *in vivo* during P. aeruginosa lung infection.

**FIG 3:**
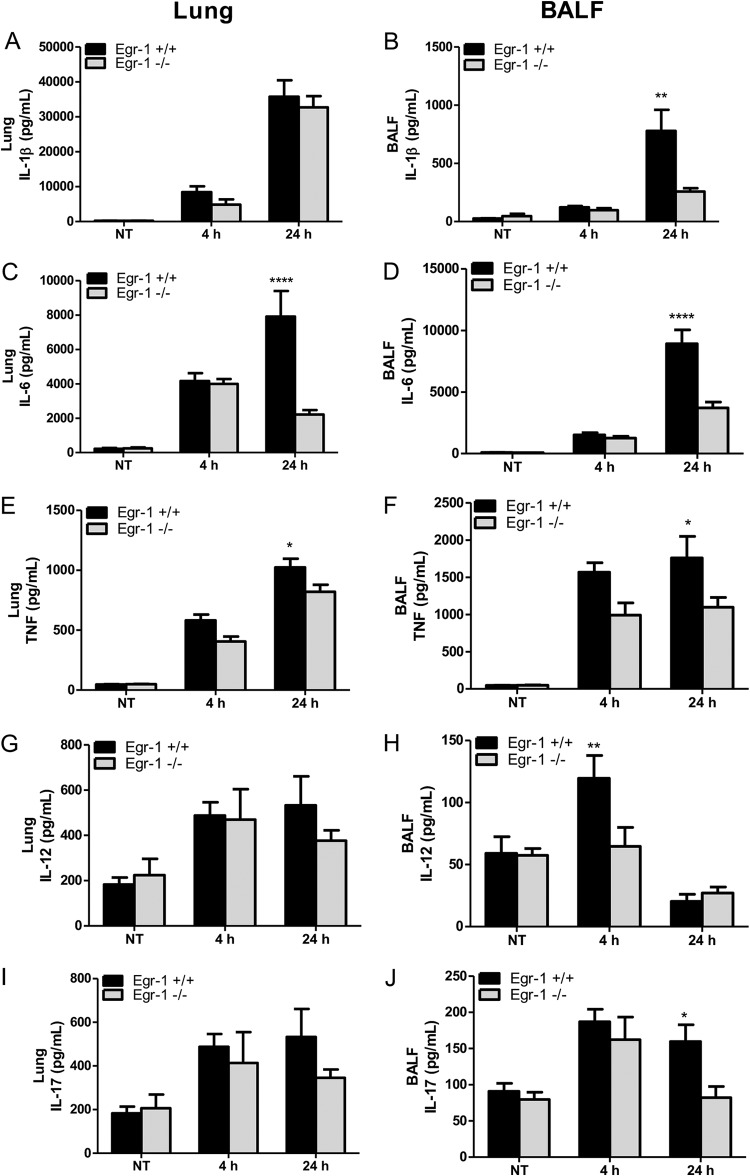
Egr-1-deficient mice displayed impaired proinflammatory cytokine production following P. aeruginosa lung infection. Wild-type (+/+) and Egr-1-deficient (−/−) mice were intranasally infected with 1 × 10^9^ CFU/mouse of P. aeruginosa 8821 for 4 h or 24 h or with an equivalent volume of saline solution as a control (NT). Mice are sacrificed after infection time points. Lung tissues (A, C, G, E, and I) and BALF (B, D, F, H, and J) were collected for detection of IL-1β (A and B), IL-6 (C and D), TNF (E and F), IL-12 (G and H), and IL-17 (I and J) (*n* = 7 to 9 ± SEM; *, *P* < 0.05; **, *P* < 0.01; ****, *P* < 0.0001).

**FIG 4:**
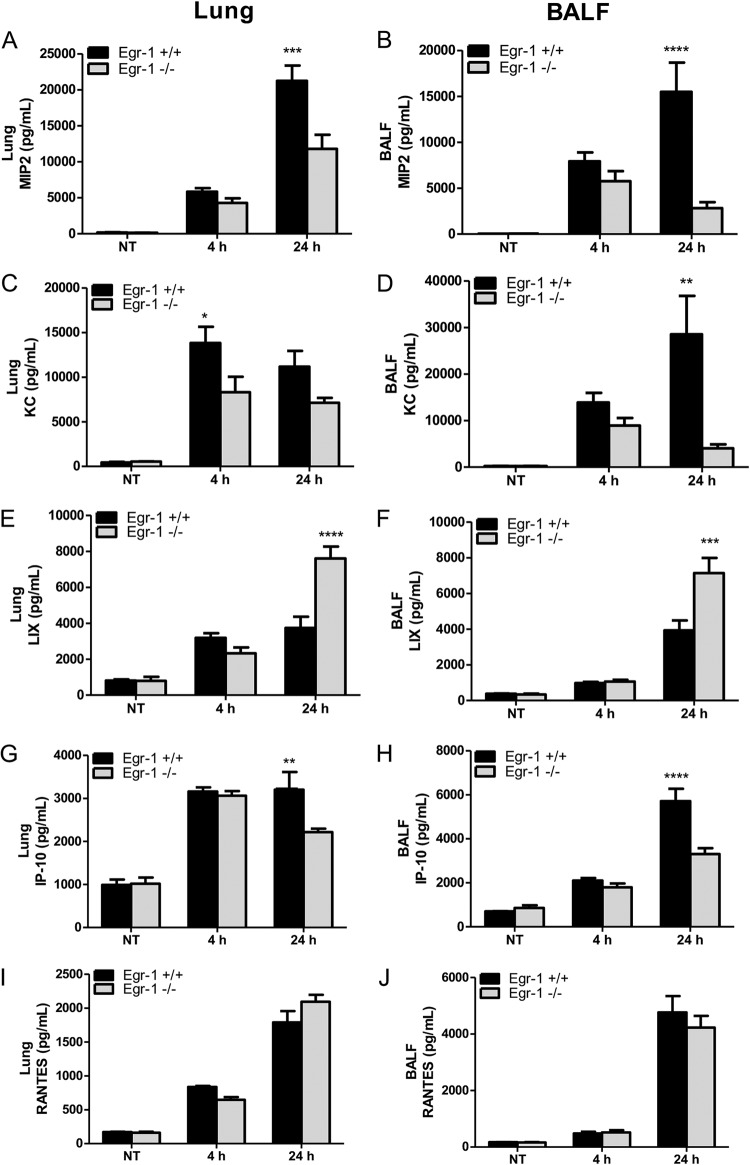
Egr-1 differentially regulates chemokine production following P. aeruginosa infection *in vivo*. Wild-type (+/+) and Egr-1-deficient (−/−) mice were intranasally infected with 1 × 10^9^ CFU/mouse of P. aeruginosa 8821 for 4 h or 24 h or with an equivalent volume of saline solution as a control (NT). Mice are sacrificed after infection time points. Lung tissues (A, C, G, E, and I) and BALF (B, D, F, H, and J) were collected for detection of MIP2 (A and B), KC (C and D), LIX (E and F), IP-10 (G and H), and RANTES (I and J) (*n* = 7 to 9 ± SEM; *, *P* < 0.05; **, *P* < 0.01; ***, *P* < 0.001; ****, *P* < 0.0001).

Egr-1 controls the production of inflammatory mediators (28). To determine whether the decreased mortality of Egr-1-deficient mice was linked to the alleviated inflammation level, we examined the *in vivo* production of proinflammatory cytokines, namely, IL-1β, IL-6, TNF, IL-12, and IL-17. In particular, IL-1β, IL-6, and TNF levels were found to be highly correlated with the intensity of the systemic inflammatory response (29). Egr-1-decifient mice showed reduced levels of IL-1β (Fig. 3A and B), IL-6 (Fig. 3C and D), TNF (Fig. 3E and F), IL-12 (Fig. 3G and H), and IL-17 (Fig. 3I and J) in lungs or BALF compared to wild-type mice during P. aeruginosa 8821 lung infection. The production of IL-1β, IL-12, and IL-17 was significantly decreased in the BALF but not in the lungs of Egr-1-deficient mice, which showed a trend of decrease but did not reach statistical significance. Furthermore, the P. aeruginosa-induced mRNA expression of IL-1β, IL-6, and TNF in lungs strongly correlated with protein levels (see Fig. S1A to C in the supplemental material). These findings suggest that Egr-1 contributes to inflammation in the P. aeruginosa-infected lung. To assess whether Egr-1 plays a role in chemokine production, we tested the *in vivo* levels of chemokines, MIP2, KC, LIX, IP-10, and RANTES. Notably, MIP2, KC, and LIX share the same chemokine receptor, CXCR2, and are essential for neutrophil recruitment in response to P. aeruginosa lung infection (30). Interestingly, Egr-1-deficient mice displayed impaired production of MIP2 (Fig. 4A and B), KC (Fig. 4C and D), and IP-10 (Fig. 4G and H) but upregulated production of LIX (Fig. 4E and F) in lungs and BALF and no significant change in production of RANTES (Fig. 4I and J) compared to wild-type mice. Moreover, the patterns of mRNA expression of MIP2, KC, LIX, IP-10, and RANTES in lungs were similar to those of their protein levels (Fig. S1D to H). These results suggest differential regulatory roles of Egr-1 in chemokine production, which may explain the modest effects of Egr-1 deficiency on neutrophil infiltration *in vivo*.

### Egr-1 deficiency diminishes proinflammatory cytokine production *in vitro* but differentially regulates chemokine production in macrophages and dendritic cells following P. aeruginosa infection.

**FIG 5:**
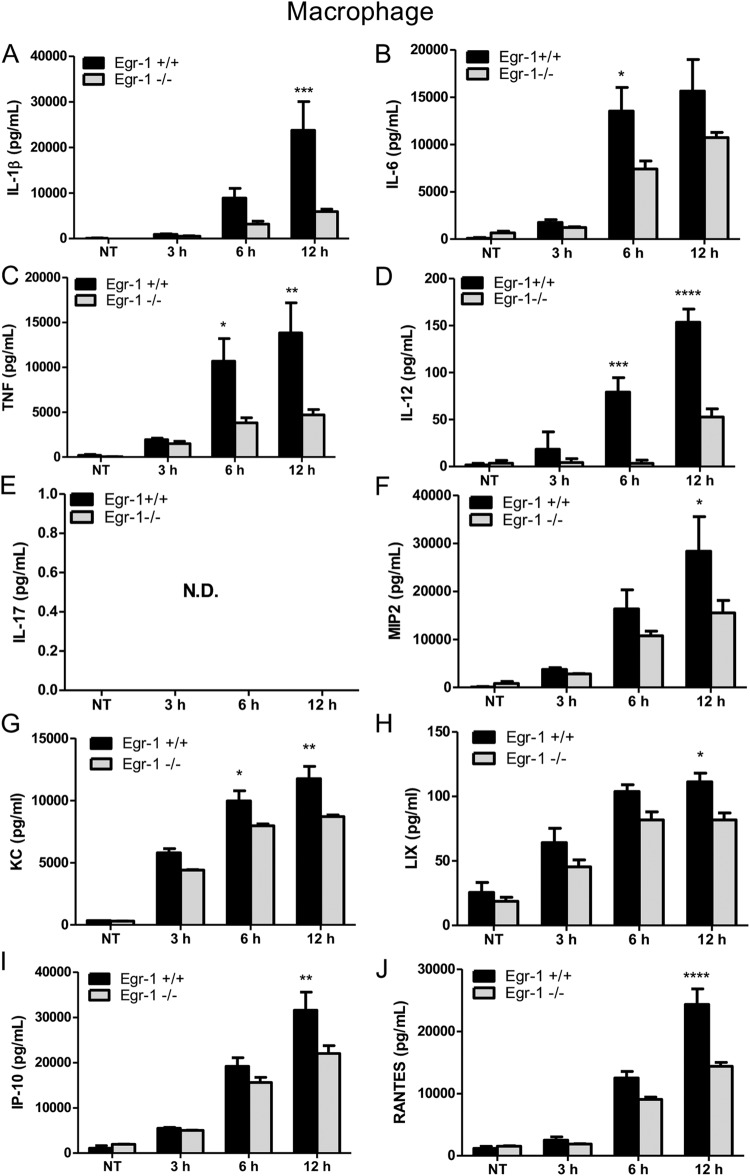
Egr-1-deficient BMMs display impaired proinflammatory cytokine and chemokine production following P. aeruginosa infection. Wild-type (+/+) and Egr-1-deficient (−/−) BMMs were infected with P. aeruginosa 8821 at an MOI of 10 for 3 h, 6 h, or 12 h or were left untreated (NT). Cell supernatants were collected for the determination of IL-1β (A), IL-6 (B), TNF (C), IL-12 (D), IL-17 (E), MIP2 (F), KC (G), LIX (H), IP-10 (I), and RANTES (J) secretion by ELISA (*n* = 3 ± SEM; *, *P* < 0.05; **, *P* < 0.01; ***, *P* < 0.001; ****, *P* < 0.0001; N.D., not detected).

Alveolar macrophages play important roles in host defense against P. aeruginosa (31). Egr-1 expression was previously shown to be induced by lipopolysaccharide (LPS) in human monocytes through a MEK–extracellular signal-regulated kinase (ERK)–Elk-1 pathway (32). To test whether P. aeruginosa induces Egr-1 expression in macrophages, wild-type bone marrow-derived macrophages (BMMs) were infected with P. aeruginosa 8821 at a multiplicity of infection (MOI) of 10 for 30 min, 1 h, 2 h, or 4 h or were left untreated. We found that Egr-1 mRNA and protein levels rapidly and transiently increased upon P. aeruginosa infection (Fig. 1). To examine the impact of Egr-1 on production of cytokines and chemokines *in vitro*, wild-type and Egr-1-deficient BMMs were infected with P. aeruginosa 8821 at an MOI of 10 for 3 h, 6 h, and 12 h. Egr-1-deficient BMMs showed diminished protein levels of IL-1β, IL-6, TNF, IL-12, MIP2, KC, LIX, IP-10, and RANTES compared to wild-type BMMs (Fig. 5). IL-17 was not detected in P. aeruginosa-infected BMMs, suggesting that macrophages are not the major source of IL-17 in response to P. aeruginosa infection *in vivo* (Fig. 5E). Similarly, the cytokine and chemokine mRNA levels were decreased in Egr-1-deficient BMMs compared to wild-type BMMs (Fig. S2). The reduced levels of LIX and RANTES production in Egr-1-deficient BMMs *in vitro* contrasted with our *in vivo* observations of increased LIX and unchanged RANTES levels in Egr-1-deficient mice, suggesting that other immune cells may contribute to the increased levels of LIX and RANTES production *in vivo* (31). Nonmucoid P. aeruginosa strain PAO1 elicited similar cytokine and chemokine responses (Fig. S3), suggesting a common role for Egr-1 in host responses to divergent P. aeruginosa isolates.

**FIG 6:**
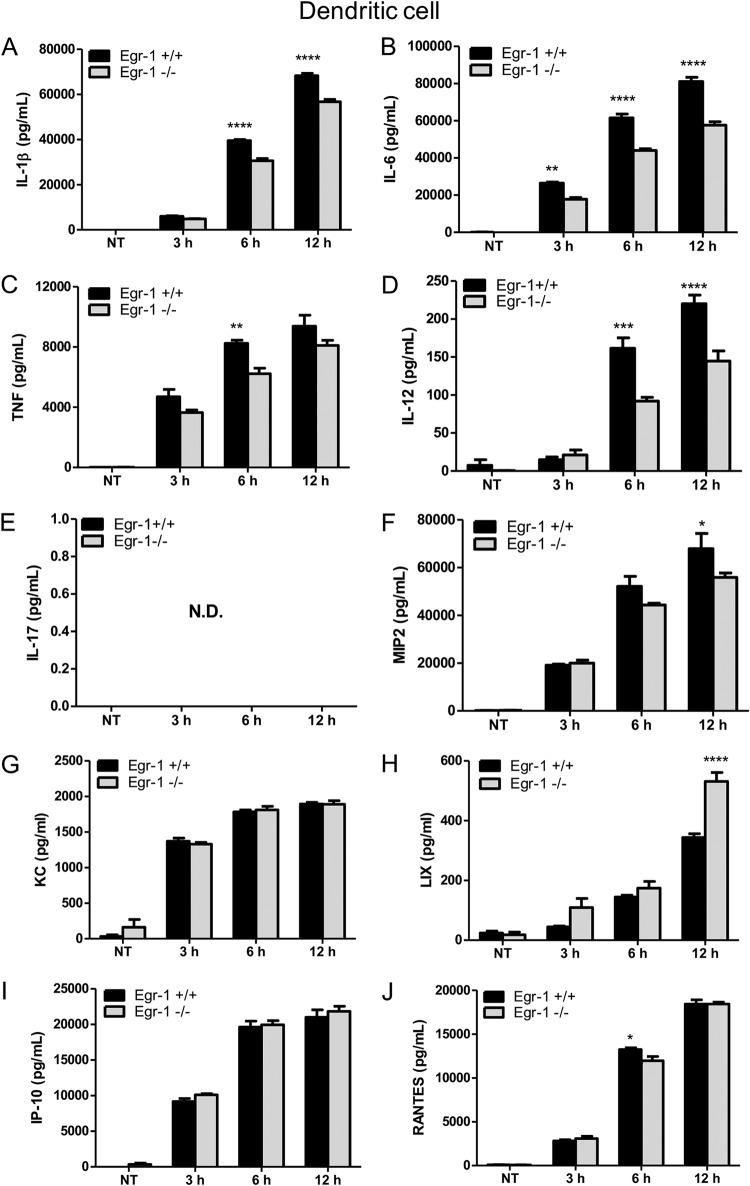
Egr-1-deficient BMDCs have increased LIX production following P. aeruginosa infection. Wild-type (+/+) and Egr-1-deficient (−/−) BMDCs were infected with P. aeruginosa strain 8821 at an MOI of 10 for 3 h, 6 h, or 12 h or were left untreated (NT). Cell supernatants were collected for the determination of IL-1β (A), IL-6 (B), TNF (C), IL-12 (D), IL-17 (E), MIP2 (F), KC (G), LIX (H), IP-10 (I), and RANTES (J) secretion by ELISA (*n* = 3 ± SEM; *, *P* < 0.05; **, *P* < 0.01; ****, *P* < 0.0001; N.D., not detected).

Dendritic cells contribute to host defenses against pulmonary P. aeruginosa infection (33, 34). Similarly to our findings in BMMs, we observed that Egr-1-deficient bone marrow dendritic cells (BMDCs) produced less TNF, IL-1β, IL-6, IL-12, MIP2, and RANTES than wild-type BMDCs during P. aeruginosa 8821 infection (Fig. 6). Interestingly, no significant difference was observed in the levels of KC and IP-10 production (Fig. 6G and I), and LIX production was markedly increased in Egr-1-deficient BMDCs compared to wild-type BMDCs (Fig. 6H). Similar responses were observed in PAO1-infected wild-type and Egr-1-deficient BMDCs, except that RANTES levels were significantly increased in Egr-1-deficient BMDCs (Fig. S4). These results suggest that Egr-1 regulates chemokine production differently in different cell types and that dendritic cells may be a major source of LIX *in vivo*.

### Egr-1 deficiency impairs NF-κB activity both *in vivo* and *in vitro* in response to P. aeruginosa infection.

**FIG 7:**
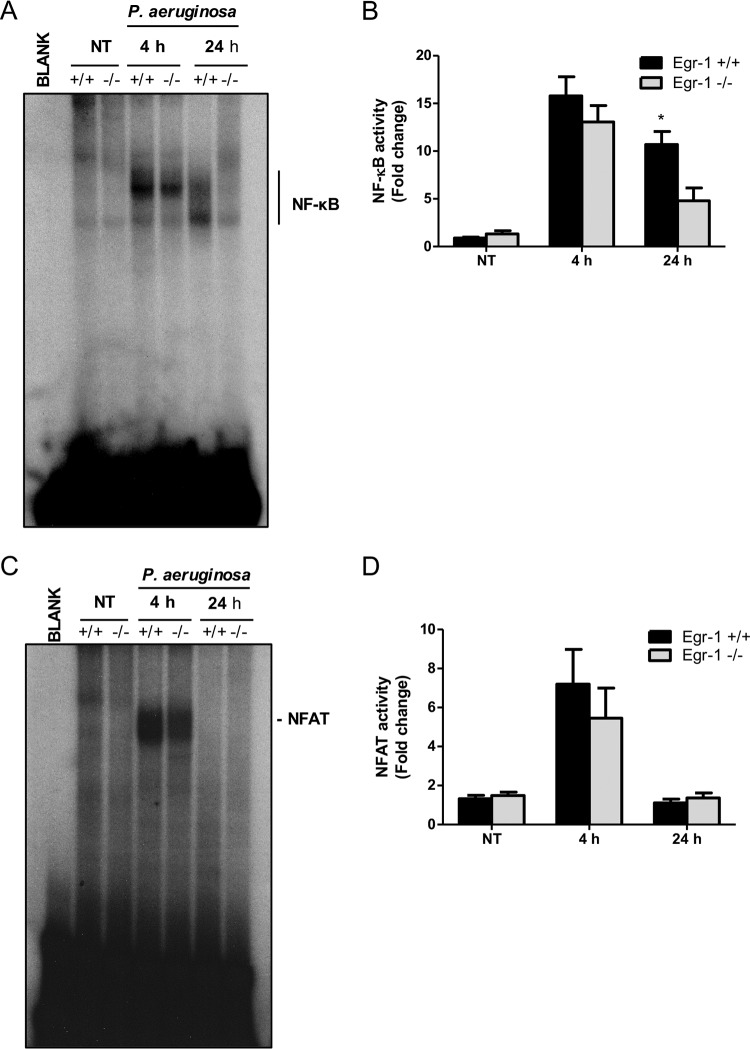
Egr-1 deficiency results in impaired NF-κB activation following P. aeruginosa infection *in vivo*. Wild-type (+/+) and Egr-1-deficient (−/−) mice were intranasally infected with 1 × 10^9^ CFU/mouse of P. aeruginosa 8821 for 4 h or 24 h or with an equivalent volume of saline solution as a control (NT). Mice are sacrificed after infection time points. Nuclear proteins were extracted and subjected to EMSA by incubation with ^32^P-labeled NF-κB (A) and NFAT (C) DNA probes. Scan densitometry was performed for analysis of NF-κB (B) and NFAT (D) activation, and data are expressed as fold change versus wild-type untreated lung (*n* = 6 ± SEM; *, *P* < 0.05).

We previously determined that transcription factors NF-κB and NFAT are essential for mediating P. aeruginosa-activated inflammatory responses (35, 36). To determine whether Egr-1 impacts NF-κB and NFAT activities *in vivo*, nuclear extracts from the lungs of P. aeruginosa 8821-infected wild-type and Egr-1-deficient mice were subjected to electrophoretic mobility shift assay (EMSA) for analysis of NF-κB and NFAT DNA-binding activities. The P. aeruginosa-induced lung NF-κB and NFAT activities peaked by 4 h postinfection. High NF-κB activity was sustained at 24 h in P. aeruginosa-infected wild-type mice, but NF-κB activity returned to nearly basal levels in Egr-1-deficient mice (Fig. 7A and B), suggesting that Egr-1 plays a role in stabilization of NF-κB DNA binding activity during P. aeruginosa infection *in vivo*. No significant differences between wild-type and Egr-1-deficient mice were observed in NFAT activity levels (Fig. 7C and D). This suggests that Egr-1 had little influence on P. aeruginosa-induced NFAT activation *in vivo*. To further analyze NF-κB and NFAT activities *in vitro*, nuclear extracts from P. aeruginosa-infected or mock-infected wild-type and Egr-1-deficient BMMs were processed for EMSA. The Egr-1-deficient BMMs had significantly reduced levels of NF-κB and NFAT activities compared to wild-type BMMs during P. aeruginosa infection (Fig. S5), suggesting that Egr-1 affects NF-κB and NFAT activation *in vitro*.

### Egr-1 has no effect on IκBα phosphorylation but physically interacts with NF-κB in macrophages in response to P. aeruginosa infection.

**FIG 8:**
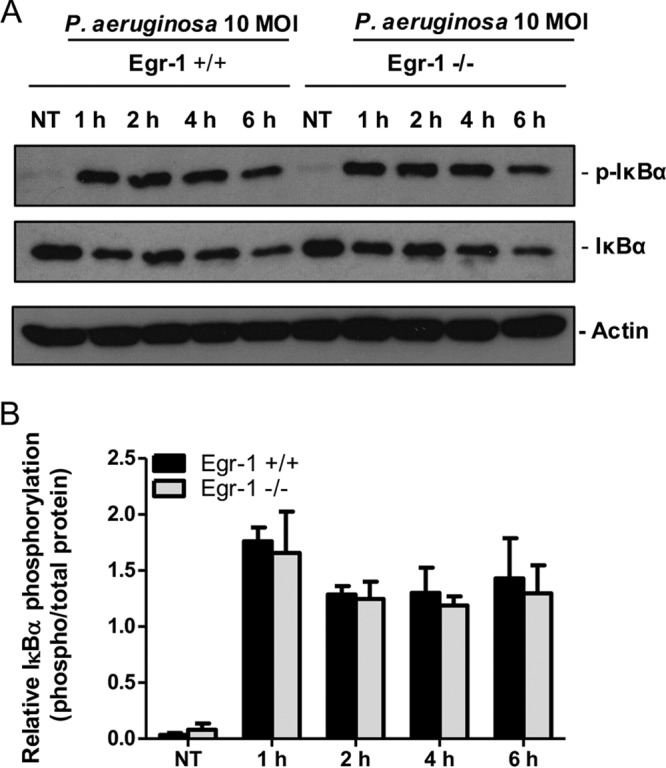
Egr-1 has no effect on IκBα phosphorylation in macrophages in response to P. aeruginosa infection. Wild-type (+/+) and Egr-1-deficient (−/−) BMMs were infected with P. aeruginosa 8821 at an MOI of 10 for 1 h, 2 h, 4 h, or 6 h or were left untreated (NT). Cell lysates were subjected to Western blotting for determining phosphorylated and total levels of IκBα, as well as of actin as a loading control. Blots are representative of three independent experiments (A). Densitometry analysis of phosphorylated IκBα was normalized to total IκBα (B) (*n* = 3 ± SEM).

**FIG 9:**
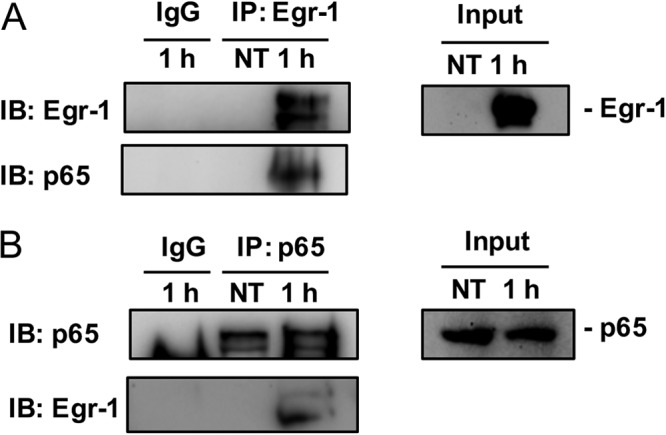
Egr-1 physically interacts with NF-κB p65 in macrophages upon P. aeruginosa infection. Wild-type (+/+) and Egr-1-deficient (−/−) BMMs were infected with P. aeruginosa 8821 at an MOI of 10 for 1 h or left untreated (NT). Cell lysates were subjected to immunoprecipitation (IP) using anti-Egr-1 (A) or anti-p65 (B) antibody followed by Western blotting for Egr-1 or p65. Mouse or rabbit IgG was used as a control. Blots are representative of three independent experiments (*n* = 3). IB, immunoblotting.

In unstimulated cells, NF-κB is sequestered and inactivated by IκBα in cytoplasm. Phosphorylation of IκBα leads to dissociation of NF-κB from IκBα, and the liberated NF-κB translocates to the nucleus, where it transactivates genes that regulate immunity, inflammation, and cell fate (37). No significant differences in IκBα phosphorylation and total IκBα levels were observed between wild-type and Egr-1-deficient BMMs during P. aeruginosa 8821 infection (Fig. 8), suggesting that IκBα is unlikely to be a direct target of Egr-1. Because mitogen-activated protein kinase (MAPK) pathways regulate inflammatory responses to P. aeruginosa infection (38), we tested the P. aeruginosa-activated phosphorylation levels of p38, ERK, and Jun N-terminal protein kinase (JNK) in macrophages. However, no significant differences were found between wild-type and Egr-1-deficient BMMs (Fig. S6). The NF-κB p65 subunit plays a critical role in regulation of inflammatory responses by binding to the promoter of inflammatory genes (39). To further assess whether Egr-1 directly affects NF-κB activation, cell lysates from P. aeruginosa-infected or untreated wild-type and Egr-1-deficient BMMs were subjected to immunoprecipitation using anti-Egr-1 or anti-NF-κB p65 antibody followed by Western blotting. The NF-κB p65 subunit was detected in the P. aeruginosa cell lysates infected for 1 h and immunoprecipitated with anti-Egr-1 antibody (Fig. 9A). Likewise, Egr-1 was detected in the P. aeruginosa cell lysates infected for 1 h in the reciprocal immunoprecipitation experiment using anti-NF-κB p65 antibody (Fig. 9B). These results suggest that Egr-1 physically interacts with the NF-κB p65 subunit without affecting IκBα phosphorylation upon P. aeruginosa infection.

### Egr-1 deficiency elevates nitric oxide production both *in vivo* and *in vitro* through enhancing expression of inducible nitric oxide synthase.

**FIG 10:**
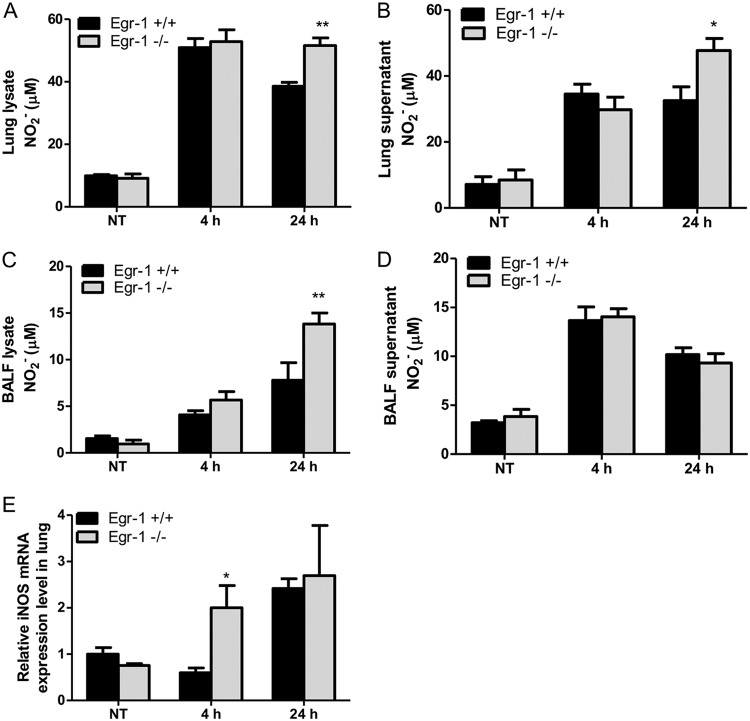
Egr-1-deficient mice show enhanced nitric oxide production and upregulated iNOS mRNA expression following P. aeruginosa lung infection. Wild-type (+/+) and Egr-1-deficient (−/−) mice were intranasally infected with 1 × 10^9^ CFU/mouse of P. aeruginosa 8821 for 4 h or 24 h or with an equivalent volume of saline solution as a control (NT). Mice are sacrificed after infection time points. The NO_2_− levels in lung lysates (A), lung supernatants (B), BALF lysates (C), and BALF supernatants (D) were assessed using a Griess reagent kit (*n* = 7 to 9 ± SEM; *, *P* < 0.05; **, *P* < 0.01). The total RNA extracted from lungs was reverse transcribed to cDNA and subjected to real-time quantitative PCR for iNOS gene expression. The gene expression was normalized to the HPRT housekeeping control gene (E) (*n* = 3 ± SEM; *, *P* < 0.05).

**FIG 11:**
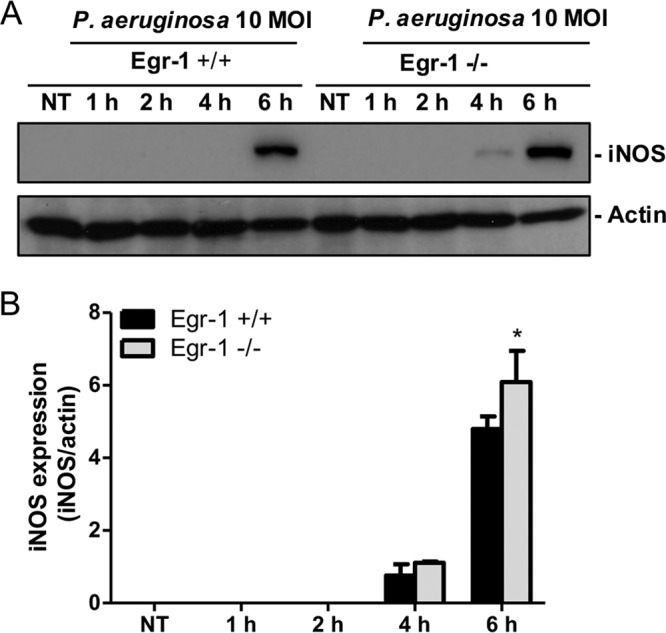
Egr-1-deficient BMMs display upregulated iNOS protein expression during P. aeruginosa infection. Wild-type (+/+) and Egr-1-deficient (−/−) BMMs were infected with P. aeruginosa 8821 at an MOI of 10 for 1 h, 2 h, 4 h, or 6 h or were left untreated (NT). Cell lysates were subjected to Western blotting for determining the protein levels of iNOS and actin as a loading control. Blots are representative of three independent experiments (A). Densitometry analysis of iNOS expression levels was normalized to actin (B) (*n* = 3 ± SEM; *, *P* < 0.05).

**FIG 12:**
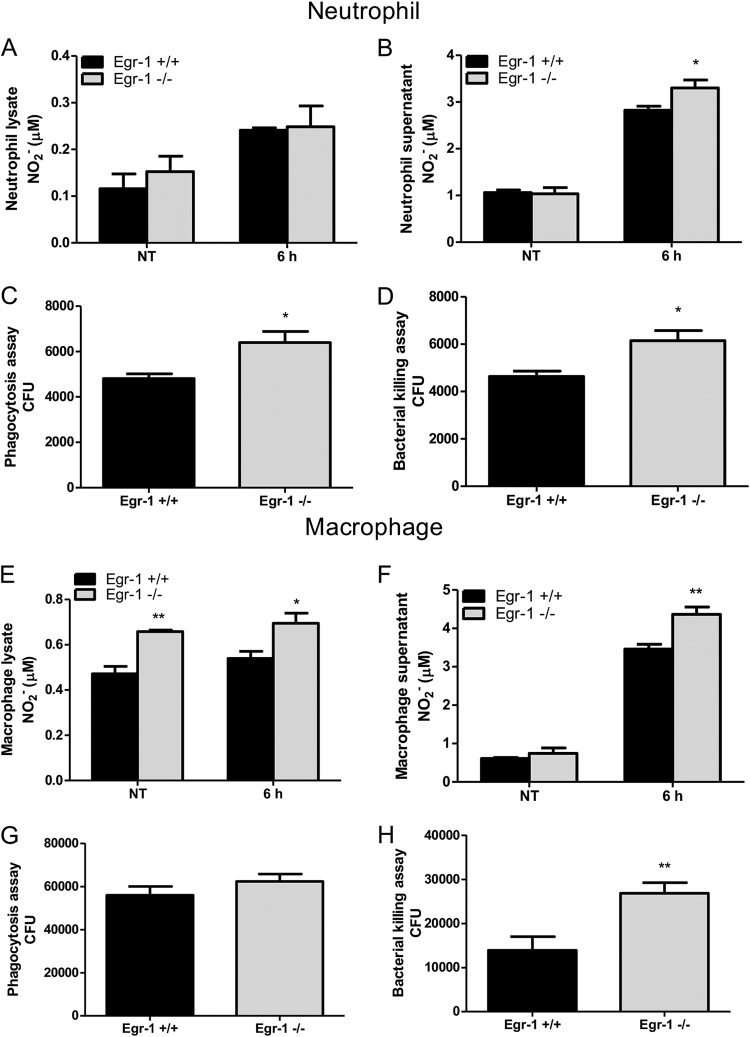
Egr-1 deficiency leads to increased nitric oxide production and enhanced bacterial intracellular levels in neutrophils and macrophages in response to P. aeruginosa infection. Wild-type (+/+) and Egr-1-deficient (−/−) neutrophils (A to D) and BMMs (E to H) were infected with P. aeruginosa 8821 for various durations. The NO_2_− levels in cell lysates (A and E) and supernatants (B and F) were determined at 6 h. The P. aeruginosa neutrophils or macrophages were infected for 1 h and lysed for phagocytosis assay (C and G). The CFU data represent the number of internalized bacteria within 1 h (*n* = 6 ± SEM; *, *P* < 0.05). The P. aeruginosa-infected neutrophils or macrophages were infected for 3 h and lysed for bacterial killing assay (D and H). The intracellular killing efficiency was calculated as the number of CFU after 1 h minus the number of CFU after 3 h of infection (1 h CFU − 3 h CFU) (*n* = 6 ± SEM; *, *P* < 0.05; **, *P* < 0.01).

Nitric oxide is a free radical produced by many types of immune cells, including macrophages and neutrophils, and it functions as a toxic defense molecule against infectious organisms (40). Importantly, nitric oxide mediates intracellular killing of P. aeruginosa in human bronchial epithelial cells (41). To analyze nitric oxide levels in lung tissues, we measured the concentration of nitrite, an oxidative product of nitric oxide, in the lysates and supernatants of lungs and BALF from wild-type and Egr-1-deficient mice. The nitrite levels in lungs and BALF were significantly increased in Egr-1-deficient mice (Fig. 10A to D). Nitric oxide production is mediated by inducible nitric oxide synthase (iNOS) in response to inflammatory stimuli (42). Egr-1-deficient mice had upregulated iNOS mRNA expression in lung at 4 h post-P. aeruginosa infection compared to wild-type mice (Fig. 10E), suggesting that upregulated iNOS expression in Egr-1-deficient mice is responsible for the increased nitric oxide production. To further assess the role of Egr-1 in nitric oxide production and iNOS expression *in vitro*, wild-type and Egr-1-deficient BMMs or neutrophils were infected with P. aeruginosa 8821 at an MOI of 10 for various durations. Egr-1-deficient BMMs showed significantly upregulated iNOS protein expression at 6 h compared to wild-type BMMs during P. aeruginosa infection (Fig. 11). Moreover, the P. aeruginosa-induced nitric oxide production was enhanced in both Egr-1-deficient neutrophils (Fig. 12A and B) and BMMs (Fig. 12E and F) compared to wild-type neutrophils and BMMs, which is consistent with the *in vivo* results. Consistently, the PAO1-infected Egr-1-deficient neutrophils and BMMs displayed elevated nitric oxide production compared to wild-type neutrophils and BMMs (Fig. S7). These findings suggest that Egr-1 deficiency increases nitric oxide production through upregulation of iNOS expression.

### Egr-1 deficiency leads to enhanced intracellular killing ability in neutrophils and macrophages.

To determine whether Egr-1 deficiency affects phagocytic activity and intracellular killing ability *in vitro*, wild-type and Egr-1-deficient neutrophils or BMMs were infected with P. aeruginosa 8821 for 1 h or 3 h. Egr-1-deficient neutrophils had enhanced phagocytic activity and intracellular killing ability compared to wild-type neutrophils (Fig. 12C and D). Furthermore, no significant differences in phagocytic activity were observed between wild-type and Egr-1-deficient BMMs, but the intracellular killing ability of Egr-1-deficient BMMs was significantly increased compared to wild-type BMMs (Fig. 12G and H). These findings demonstrate that Egr-1 has a negative impact on intracellular killing ability in neutrophils and macrophages.

### Egr-1 deficiency has no impact on P. aeruginosa-induced apoptosis in macrophages.

Because Egr-1 has been shown to regulate apoptosis (43, 44), we investigated whether Egr-1 deficiency affected P. aeruginosa-induced apoptosis *in vitro*. Wild-type and Egr-1-deficient BMMs were infected with P. aeruginosa 8821 at an MOI of 10 for 2 h or were left untreated and were processed for measurement of apoptosis by flow cytometry (Fig. S8A to D). P. aeruginosa infection caused similar percentages of cell death in wild-type and Egr-1-deficient BMMs (Fig. S8E). Interestingly, P. aeruginosa infection slightly increased the number of apoptotic wild-type BMMs but slightly reduced that of apoptotic Egr-1-deficient BMMs. However, no significant difference was found in the numbers of apoptotic wild-type and Egr-1-deficient BMMs (Fig. S8F). This finding suggests that Egr-1 deficiency has no impact on macrophage apoptosis during P. aeruginosa infection.

## DISCUSSION

**FIG 13:**
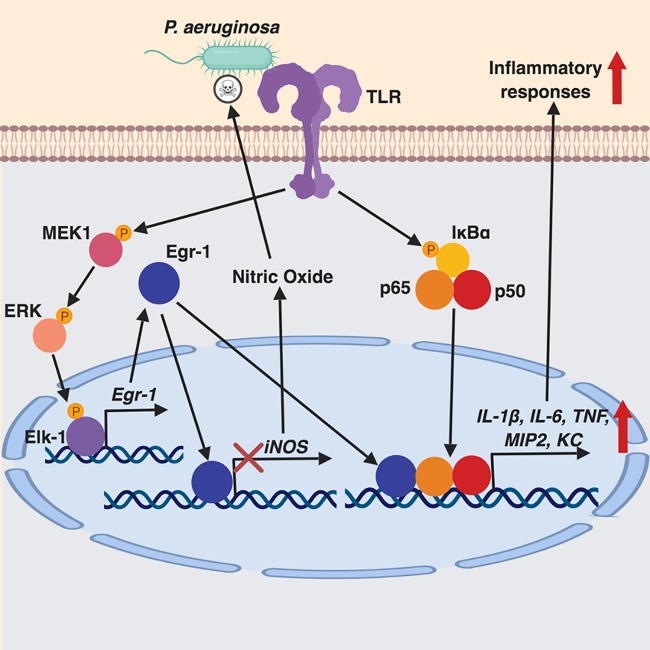
Schematic representation of Egr-1-regulated host defense against P. aeruginosa infection. Egr-1 negatively regulates nitric oxide production by suppressing iNOS gene expression and promotes inflammatory cytokine production by physically interacting with NF-κB p65 during P. aeruginosa infection.

Host inflammatory responses to P. aeruginosa infection are regulated by multiple signaling pathways, including MyD88-NF-κB, NFAT, and MAPK pathways (35, 36, 45). These inflammatory responses aid bacterial clearance, but excessive inflammation leads to persistent P. aeruginosa infection and lung damage (46). The molecular mechanisms involved in regulation of inflammation during P. aeruginosa infection are not fully understood. The Egr-1 transcription factor governs many cellular processes and inflammation (10–13, 28). Aberrant Egr-1 expression has been implicated in pulmonary inflammation (47). In this study, we identified upregulated Egr-1 expression in response to P. aeruginosa infection and characterized a detrimental role of Egr-1 in host defense against this bacteria mediated by promoting inflammation and negatively regulating nitric oxide production (Fig. 13).

The intensity of systemic inflammatory response is reflected in the levels of proinflammatory cytokines IL-1β, IL-6, and TNF (29). IL-12 was shown to contribute to host defense against P. aeruginosa infection by inducing IFN-γ production (48). IL-17 is primarily produced by Th17 cells, and it induces production of other cytokines and chemokines such as IL-6, granulocyte-macrophage colony-stimulating factor (GM-CSF), MIP2, KC, LIX, and MCP1, promoting recruitment of neutrophils and macrophages (49). Although robust inflammatory responses normally aid bacterial clearance, sustained systemic inflammatory responses and organ failure as consequences of persistent P. aeruginosa infection can be life-threatening (6). Previous studies have shown that Egr-1 contributed to production of inflammatory mediators in response to stimulation of bacterial cell wall components such as LPS and peptidoglycan (28, 50). Our data showed that Egr-1-deficient mice had reduced but not abolished levels of IL-1β, IL-6, TNF, IL-12, and IL-17 compared to wild-type mice during P. aeruginosa lung infection and that those results were associated with decreased mortality. The production of IL-1β, IL-6, and TNF is primarily mediated through the MyD88-NF-κB pathway during P. aeruginosa infection (35). Activation of the MyD88-NF-κB pathway leads to IκBα phosphorylation and NF-κB activation (37). Egr-1 deficiency had no effect on IκBα phosphorylation but significantly impaired NF-κB activation following P. aeruginosa infection. This finding suggests that Egr-1 directly affects NF-κB activity but not its upstream signaling. Previous studies have shown that Egr-1 interacted with other transcription factors and cooperatively regulated transcription of proinflammatory cytokine genes (22, 23). The interaction between Egr-1 and the other transcription factors results in formation of stable heterodimeric protein complexes, which promotes DNA binding and transcription activities. Here, we provided strong evidence showing that Egr-1 physically interacted with NF-κB p65 upon P. aeruginosa infection, leading to a prolonged period of NF-κB activation. Additionally, we previously reported that NFAT mediated inflammatory gene expression through cross talk with NF-κB during P. aeruginosa infection (36). However, our data showed that Egr-1 deficiency had a limited impact on NFAT activation *in vivo*.

Strong inflammatory responses are usually associated with enhanced bacterial clearance. However, our findings demonstrate that Egr-1 deficiency caused enhanced bacterial clearance without affecting neutrophil and macrophage infiltration, suggesting that the bacterial clearance may be mediated through other mechanisms. Nitric oxide is a potent antimicrobial agent that is produced from l-arginine by NOS enzymes, namely, iNOS, neuronal NOS (nNOS), and endothelial NOS (eNOS) (51). Isoforms nNOS and eNOS are constitutively expressed and produce low level of nitric oxide, whereas iNOS is induced in many types of cells by inflammatory stimuli and produces large amount of nitric oxide (52). Importantly, nitric oxide synthesized from iNOS is important for combating P. aeruginosa infection (41). Our results revealed that Egr-1 deficiency led to upregulated iNOS expression and nitric oxide production, which was found to be associated with enhanced intracellular killing ability in neutrophils and macrophages. These results could explain the enhanced bacterial clearance in Egr-1-deficienct mice even though there were no differences in the numbers of recruited neutrophils or macrophages. Egr-1 can positively or negatively regulate target gene expression (19). Consistent with our findings, a previous study demonstrated that P2X7-dependent Egr activation decreased iNOS expression and NO production whereas it increased TNF-α production and that the Egr DNA binding sites were identified in the iNOS promoter (53). The phagocytic activity of neutrophils and macrophages has been found to be essential for P. aeruginosa clearance *in vivo* (27, 54–56). In this study, we identified an elevated level of phagocytic activity in Egr-1-deficienct neutrophils, which may also contribute to bacterial clearance. A previous study reported that suppression of Egr-1 expression and nuclear translocation by β-defensins 2 and 3 enhanced expression of phagocytic receptors and promoted phagocytosis of P. aeruginosa (57). Moreover, Egr-1 was previously reported to promote autophagy, which is able to suppress phagocytic activity (58, 59). Hubbard et al. found that the alveolar macrophages from bone-marrow-transplanted mice displayed diminished (∼30% of normal) phagocytic activity in response to P. aeruginosa infection, which correlated with an approximately 3.5-times-higher bacterial burden in the lungs than in wild-type mice (56). Similarly, our data showed that Egr-1-deficient neutrophils had increased phagocytic activity (∼30% more) (Fig. 12C) and that Egr-1-deficient mice displayed an ∼4.5-fold decrease of pulmonary bacterial burden compared to wild-type mice (Fig. 2C).

Apoptosis is a form of programmed cell death that plays defensive roles to clear bacterial pathogens without eliciting an inflammatory response (60). However, apoptosis also benefits bacterial pathogens, eliminating essential immune cells and subverting normal host immune responses (61). Our data showed that Egr-1 deficiency did not significantly influence the P. aeruginosa-induced apoptosis in macrophages (see Fig. S8 in the supplemental material), suggesting that Egr-1 has limited impact on apoptosis in the context of P. aeruginosa infection.

Chemokines MIP2, KC, LIX, IP-10, and RANTES aid neutrophil recruitment during P. aeruginosa lung infection (30, 62). Indeed, our data showed that Egr-1 influenced production of these chemokines by macrophages and dendritic cells. Notably, Egr-1-deficient macrophages displayed reduced LIX production whereas the Egr-1-deficient dendritic cells had increased LIX production. However, comparing the peak values (12 h) of LIX concentrations between these two types of cells, 1.5 × 10^6^ Egr-1-deficient dendritic cells produced approximately 5-fold-higher LIX concentrations than were produced by the same number of Egr-1-deficient macrophages, suggesting that dendritic cells may be the major source of LIX *in vivo* and that the increased LIX level may compensate for the effects caused by reduced MIP2, KC, and IP-10 production. Additionally, Egr-1 deficiency had no effect on RANTES production *in vivo*. Thus, Egr-1 deficiency had no effect on neutrophil recruitment, which is consistent with a previous study showing that Egr-1 deficiency had no impact on leukocyte recruitment to lung in a mouse endotoxemia model (28). The differential chemokine production patterns between macrophages and dendritic cells can be explained by the fact that macrophages and dendritic cells respond differently to the same stimuli due to their differentially expressed receptors and distinct forms of signaling transduction (63–66). A previous study performed by Werling et al. demonstrated that macrophages and dendritic cells had differential levels of cytokine production after exposure to the same Toll-like receptor (TLR) ligands or bacterial stimuli (63). Furthermore, Sallusto et al. showed that macrophages did not produce the chemokines TARC (CCL17) and ELC (CCL19) but that these two chemokines were produced by dendritic cells in response to LPS or bacterial stimulation (66).

In this study, we used two different P. aeruginosa strains, 8821 and PAO1, to examine the levels of proinflammatory cytokine and chemokine production in BMMs and BMDCs. It is noteworthy that the PAO1-induced cytokine and chemokine levels (Fig. S3 and S4) were much lower than the levels induced by P. aeruginosa 8821 (Fig. 5 and 6). This might be explainable by the fact that P. aeruginosa mucoid strains are generally less virulent than P. aeruginosa nonmucoid strains, which is thought to be due to differences in the levels of production of virulence factors (67, 68). Previous studies have shown that P. aeruginosa virulence factors cause cell death and disrupt cellular functions (69–72). Thus, the high cytotoxicity of the nonmucoid PAO1 strain may prevent full induction of host inflammatory responses.

Altogether, our findings demonstrate a novel Egr-1 regulatory mechanism during P. aeruginosa infection, which promotes inflammatory responses by physically interacting with NF-κB and negatively regulating nitric oxide production by suppressing iNOS expression. Our results suggest that molecules that inhibit Egr-1 may increase clearance of P. aeruginosa and lower the risk of pathogenic systemic inflammation. Several Egr-1 inhibitors, including the antitumor drug mithramycin A, which inhibits radiation-induced apoptosis by preventing Egr-1 binding to target promoters (44), and metformin, which suppresses Egr-1 expression in mesangial cells through inhibition of miR-34a (73), have already been described. We speculate that metformin could be an attractive candidate to treat P. aeruginosa lung infection due to ease of administration and excellent safety profile. However, the use of Egr-1 inhibitors in clinical practice remains a work in progress.

## MATERIALS AND METHODS

### Animals.

Heterozygous Egr-1 mice (+/−) with a C57BL/6 genetic background were purchased from The Jackson Laboratories (Bar Harbor, ME, USA). Heterozygous breeders were used to establish separate wild-type (+/+) and Egr-1-deficient (−/−) breeding colonies, which were maintained in the same specific-pathogen-free facility. All animal protocols were approved by the University Committee on Laboratory Animals, Dalhousie University, in accordance with guidelines of the Canadian Council on Animal Care (protocol number 18-002).

### Antibodies.

Antibodies for Egr-1 (4153), iNOS (13120), phospho-IκB (2859), total IκB (9242), phospho-ERK (9101), total ERK (9102), phospho-p38 (9211), total p38 (8690), phospho-JNK (9251), and total JNK (9252) were purchased from Cell Signaling Technologies. Antibodies for NF-κB p65 (sc-109) and all secondary antibody conjugates were purchased from Santa Cruz Biotechnology. Antibody for actin (MA5-15739) was purchased from Thermo Fisher Scientific. Antibody for PerCP-Cy5.5 anti-mouse Ly6G (560602) was purchased from BD Biosciences. Antibody for allophycocyanin (APC) anti-mouse F4/80 (17-4801-82) was purchased from eBioscience. Brilliant violet 650 anti-mouse CD45 (103151) and fluorescein isothiocyanate (FITC) anti-mouse CD64 (139316) were purchased from BioLegend.

### Bacterial preparation.

Pseudomonas aeruginosa strain 8821, a mucoid strain isolated from a cystic fibrosis patient, was a gift from Ananda M. Chakrabarty, University of Illinois, Chicago, IL. Pseudomonas aeruginosa PAO1, a wound isolate, is one of the most commonly used laboratory strains (74). Pseudomonas aeruginosa was cultured as described previously (75). Briefly, suspension cultures were grown in Luria-Bertani (LB) broth overnight at 37°C and shaken at 225 rpm in a shaking incubator (New Brunswick Scientific Innova 4080) until the early stationary phase (optical density [OD] value at 600 nm of between 2.5 to 3). Bacteria were washed in phosphate-buffered saline (PBS) and resuspended in saline solution for *in vivo* experiments or in PBS for *in vitro* assays.

### Cell culture and P. aeruginosa infection.

For macrophage culture, bone marrow cells were flushed from femurs and tibias of wild-type (+/+) and Egr-1-deficient (−/−) mice. Cells were cultured in DMEM (Dulbecco’s modified Eagle medium) supplemented with 10% fetal bovine serum, 100 U/ml penicillin-streptomycin, and 30% L929 supernatant containing macrophage colony-stimulating factor (M-CSF). For dendritic cell culture, bone marrow cells were cultured in RPMI 1640 media supplemented with 10% fetal bovine serum, 100 U/ml penicillin-streptomycin, and 5% X-63 supernatant containing granulocyte-macrophage colony-stimulating factor (GM-CSF). Media were changed every 2 to 3 days by replacing half of the initial volume. After 7 days, cells were infected with P. aeruginosa 8821 at a multiplicity of infection (MOI) of 10 or were mock infected. At different time points postinfection, cell-free supernatants were collected for measurement of cytokine and chemokine production whereas cell pellets were processed for measurement of levels of RNAs or proteins by real-time quantitative PCR (RT-qPCR) or immunoblotting, respectively. A portion of cell pellets was reserved for transcription factor activation analysis by electrophoretic mobility shift assay (EMSA). Infections of macrophage and dendritic cells with P. aeruginosa PAO1 were performed as described above, except that only the cell-free supernatants were collected for measurement of cytokine and chemokine production.

### Lung infection with P. aeruginosa and collection of lung and bronchoalveolar lavage fluid (BALF).

Mice were infected intranasally with 1 × 10^9^ CFU of P. aeruginosa 8821 for 4 or 24 h. BALF was collected by lavaging the lungs 3 times with 1 ml PBS containing soybean trypsin inhibitor (100 μg/ml). To remove the intrapulmonary blood from lung tissues, 10 ml PBS was infused to the right atrium using a butterfly needle, and the lungs turned from pink to white. Lung tissues were homogenized in 50 mM HEPES buffer (4 μl/mg lung) containing soybean trypsin inhibitor (100 μg/ml). For counting bacterial CFU, 10-μl volumes of lung homogenates were serially diluted and plated onto an LB agar dish and incubated for 24 h at 37°C. The remaining lung homogenates were centrifuged at 4°C for 20 min at 18,000 × *g*. Supernatants were stored at −80°C for later cytokine analysis and nitric oxide assay. Pellets were resuspended and homogenized in 0.5% cetyltrimethylammonium chloride (4 μl/mg lung) and centrifuged as described above. Cleared supernatants were used for nitric oxide assay. BALF (10 μl) was serially diluted and plated onto an LB agar dish and incubated for 24 h for CFU counting. The remaining BALF was centrifuged at 480 × *g* for 5 min at 4°C. The supernatants were used for cytokine and nitric oxide analysis. Pellets were resuspended in 1 ml NH_4_Cl (0.15 M) and centrifuged as described above to lyse red blood cells. Supernatants were discarded, and the pellets were resuspended in 0.5% cetyltrimethylammonium chloride (250 μl/mouse) and then centrifuged. Cleared supernatants were used for nitric oxide assay.

### Animal survival and disease scores.

Ten wild-type and 10 Egr-1-deficient mice were infected intranasally with 1 × 10^9^ CFU of strain 8821 per mouse to measure animal survival and disease progression. Disease scores were recorded for 10 days according to the following scoring system (total possible score, 21): (i) physical appearance (0, normal; 1, lack of grooming; 2, rough hair coat; 3, very rough hair coat); (ii) posture (0, normal; 1, sitting in hunched position; 2, hunched posture, head resting on floor; 3, lying prone on cage floor/unable to maintain upright posture); (iii) activity and behavior (0, normal activity and behavior; 1, somewhat reduced activity and minor changes in behavior; 2, somewhat reduced activity and minor changes in behavior plus change in respiratory rate or effort; 3, movement only when stimulated); (iv) appetite (0, normal; 1, reduced appetite; 2, no eating since last check point; 3, no eating for last two check points); (v) hydration (0, normal; 1, mild dehydration; 2, moderate dehydration; 3, severe dehydration); (vi) body weight (0, <5% change from preinfection weight; 1, <10% weight change; 2, approximately 10% to 15% weight change; 3, approximately 15% to 19% weight change); and (vii) body temperature (ventral surface temperature) (0, approximately 33°C to 34°C; 1, approximately 28°C to 32.5°C; 2, approximately 25°C to 27.5°C; 3, <24.5°C). Animals were euthanized when the score reached 15.

### Cytokine production.

Concentrations of IL-1β, IL-6, TNF, IL-12, IL-17, MIP2, KC, LIX, IP-10, and RANTES in lungs, BALF, and culture supernatants were determined by enzyme-linked immunosorbent assay (ELISA) as described previously (75) using antibody pairs from R&D Systems (Minneapolis, MN).

### Real-time quantitative PCR.

Cells were processed using TRIzol (Invitrogen), and RNA was purified using an RNeasy kit (Qiagen). The total RNA was reverse transcribed into cDNA using RNA corresponding to cDNA EcoDry Premix (TaKaRa Bio). The *egr-1* primer sequences were as follows: forward, 5′-CCGCTTTTCTCGCTCGGATG-3′; reverse, 5′-GCGGATGTGGGTGGTAAGGT-3′. The *iNOS* primer sequences were as follows: forward, 5′-CAGCTGGGCTGTACAAACCTT-3′; reverse, 5′-CATTGGAAGTGAAGCGTTTCG-3′. The *IL-1β* primer sequences were as follows: forward, 5′-TGCCACCTTTTGACAGTGATGA-3′; reverse, 5′-TGCCTGCCTGAAGCTCTTGT-3′. The *IL-6* primer sequences were as follows: forward, 5′-TAGTCCTTCCTACCCCAATTTCC-3′; reverse, 5′-TTGGTCCTTAGCCACTCCTTC-3′. The *TNF* primer sequences were as follows: forward, 5′-CATCTTCTCAAAATTCGAGTGACAA-3′; reverse, 5′-TGGGAGTAGACAAGGTACAACCC-3′. The *MIP2* primer sequences were as follows: forward, 5′-CCACTCTCAAGGGCGGTCAA-3′; reverse, 5′-GGTACGATCCAGGCTTCCCG-3′. The *KC* primer sequences were as follows: forward, 5′-TGCAGACCATGGCTGGGATT-3′; reverse, 5′-AGCCTCGCGACCATTCTTGA-3′. The *LIX* primer sequences were as follows: forward, 5′-GCGGTTCCATCTCGCCATTC-3′; reverse, 5′-TCCGTTGCGGCTATGACTGA-3′. The *IP-10* primer sequences were as follows: forward, 5′-ATCATCCCTGCGAGCCTATCCT-3′; reverse, 5′-GACCTTTTTTGGCTAAACGCTTTC-3′. The *RANTES* primer sequences were as follows: forward, 5′-CCTGCTGCTTTGCCTACCTCTC-3′; reverse, 5′-ACACACTTGGCGGTTCCTTCGA-3′. The primers were designed by Primer-BLAST (NCBI). Real-time quantitative PCR (RT-qPCR) assays were conducted in triplicate using the SYBR green method on a CFX Connect real-time PCR detection system (Bio-Rad) according to manufacturer’s instructions. Hypoxanthine-guanine phosphoribosyltransferase (HPRT) was used as housekeeping control mRNA. Data were analyzed using the relative standard curve method according to the manufacturer’s protocol.

### Western blotting.

Cells were lysed in radioimmunoprecipitation assay (RIPA) buffer (Sigma-Aldrich; catalog no. R0278) supplemented with a mixture of protease and phosphatase inhibitors. Cleared lysates (30 μg protein) were electrophoresed in 10% SDS-polyacrylamide gels. Gels were transferred to a polyvinylidene difluoride membrane (Bio-Rad), blocked with 5% nonfat milk, probed with primary and secondary antibodies, and detected by an ECL detection system (Western Lightning Plus-ECL; PerkinElmer) on BioMax film (Kodak). Blots were scanned and quantified using ImageJ software.

### Immunoprecipitation.

Bone marrow-derived macrophages (BMMs) were infected with P. aeruginosa 8821 at an MOI of 10 for 1 h or left untreated. Cell lysates (500 μg) were incubated with 20 μl protein A/G-linked agarose and 2 μl IgG for 30 min at 4°C to exclude nonspecific binding. The precleared lysates were incubated with 1 μg of anti-Egr-1 or anti-NF-κB p65 antibody overnight at 4°C. The next day, the samples were incubated with 20 μl protein A/G agarose beads for 2 h at 4°C. IgG was used as the negative control. Beads were washed four times with 500 ml RIPA buffer after incubation and dissolved in 2× SDS-PAGE loading buffer. Subsequent steps were performed as described above under “Western blotting.”

### Flow cytometry.

Cells extracted from mouse lung tissues and BALF were resuspended in 0.15 M NH_4_Cl buffer to lyse erythrocytes and were stained with Fixable Viability Stain (FVS) 510 (BD Biosciences) at a 1:1,000 dilution in PBS at 4°C for 20 min. Cells were washed and resuspended in PBS–1% bovine serum albumin (BSA) and were incubated with Fc block (BD Biosciences) for 15 min at 4°C. After blocking, the cells were stained with the antibodies for Ly6G, CD45, F4/80, and CD64 at a 1: 200 dilution for 30 min at 4°C. The cells were then fixed with 1% paraformaldehyde for 20 min at 4°C and resuspended in PBS–1% BSA. Antibody-stained cells were acquired on a CytoFLEX flow cytometer (Beckman Coulter), and the data were analyzed using FCS Express 6 flow cytometry software (De Novo Software). The gating strategies used for neutrophils and macrophages were described in a previous study (76). Neutrophils were characterized as CD45^+^, F4/80^−^, and Ly6G^+^. Macrophages were characterized as CD45^+^, F4/80^+^, CD64^+^, and Ly6G^−^.

For apoptosis analysis, wild-type and Egr-1-deficient BMMs were infected with P. aeruginosa 8821 at an MOI of 10 for 2 h or left untreated. The cells were stained with FITC-annexin V (BD Biosciences) at a 1:50 dilution and with the viability dye 7-aminoactinomycin D (7-AAD) (Molecular Probes) at a 1:200 dilution for 20 min at 4°C. The cells were acquired by flow cytometry as described above. Apoptotic cells were defined as cells that were either annexin V positive only or annexin V and 7-AAD positive, while dead cells were defined as cells that were positive only for 7-AAD.

### Measurement of nitric oxide production.

Neutrophils were isolated from mouse bone marrow using a mouse neutrophil enrichment kit (Stemcell Technologies Inc.). Neutrophils and macrophages were infected with P. aeruginosa strain 8821 or PAO1 at an MOI of 10 for 6 h or left untreated. Cell-free supernatants and cell lysates were collected for measurement of the extracellular and intracellular nitric oxide levels, respectively, using a Griess reagent kit (Thermo Fisher Scientific).

### Phagocytosis and intracellular killing assay.

Phagocytosis and intracellular killing assays were described previously (77). Macrophages or neutrophils were infected with P. aeruginosa 8821 at an MOI of 10. For the phagocytosis assay, cells were collected to enumerate internalized bacteria after 1 h of incubation. For intracellular killing assay, 100 μg/ml gentamicin was added to the cell culture medium after 1 h postinfection to eliminate the extracellular bacteria. Cells were incubated for another 2 h to evaluate the intracellular killing of bacteria. For both the phagocytosis and intracellular killing assays, cells were then washed with PBS, pelleted, and lysed with 0.1% Triton X-100. Cell lysates were serially diluted and plated on LB agar plates for CFU counting. Phagocytic activity was analyzed based on the CFU data obtained 1 h after infection. Intracellular killing efficiency was calculated as the number of CFU at 3 h postinfection subtracted from the number of CFU at 1 h postinfection (1-h CFU – 3-h CFU).

### Nuclear extract preparation and electrophoresis mobility shift assay (EMSA).

EMSA was performed as previously described (35). Briefly, nuclear protein extracts were prepared using a nuclear extract kit (Active Motif, Carlsbad, CA), following the manufacturer’s protocol. Probe labeling was accomplished by treatment with T4 kinase (Life Technologies, Burlington, ON, Canada) in the presence of [^32^P]ATP (Perkin Elmer, Waltham, MA). Labeled oligonucleotides were purified on a Sephadex G-25M column (GE Healthcare, Pittsburgh, PA). Nuclear protein (10 μg) was added to a 10-μl volume of binding buffer supplemented with 1 μg poly(dI:dC) (GE Healthcare) for 15 min at room temperature. Labeled double-stranded oligonucleotide was added to each reaction mixture, incubated at room temperature for 30 min, and separated by electrophoresis on a 6% polyacrylamide gel in 0.5× Tris-boric acid-EDTA buffer. Gels were vacuum-dried and subjected to autoradiography. The following synthesized double-stranded oligonucleotides were used: NF-κB consensus sequence on the IL-6 promoter (5′-AGTTGAGGGGACTTTCCCAGGC-3′) (Promega, Madison, WI) and NFAT-binding consensus sequence on the mouse IL-13 promoter (5′-AAGGTGTTTCCCCAAGCCTTTCCC-3′) (Sigma-Aldrich).

### Statistical analysis.

Data are presented as means ± standard errors of the means (SEM) of the indicated number of experiments. The statistical significance of results of comparisons between multiple treatments was determined by one-way analysis of variance and *post hoc* Tukey’s honest significance test. Alternatively, for analyses of two independent variables, a two-way analysis of variance and a Bonferroni multiple-comparison test were used.

Statistical analysis was performed using GraphPad Prism software version 5.04 (GraphPad Software Inc., La Jolla, CA).

## Supplementary Material

Supplemental file 1
