# Supplementary material for: Early Growth Response 1 Deficiency Protects the Host against Pseudomonas aeruginosa Lung Infection
Source: Infect Immun. 2019 Dec 17;88(1):e00678-19. doi: 10.1128/IAI.00678-19 (PMC6921661; doi:10.1128/IAI.00678-19)
Supplement: Supplemental file 1 [file IAI.00678-19-s0001.pdf]

Fig S1

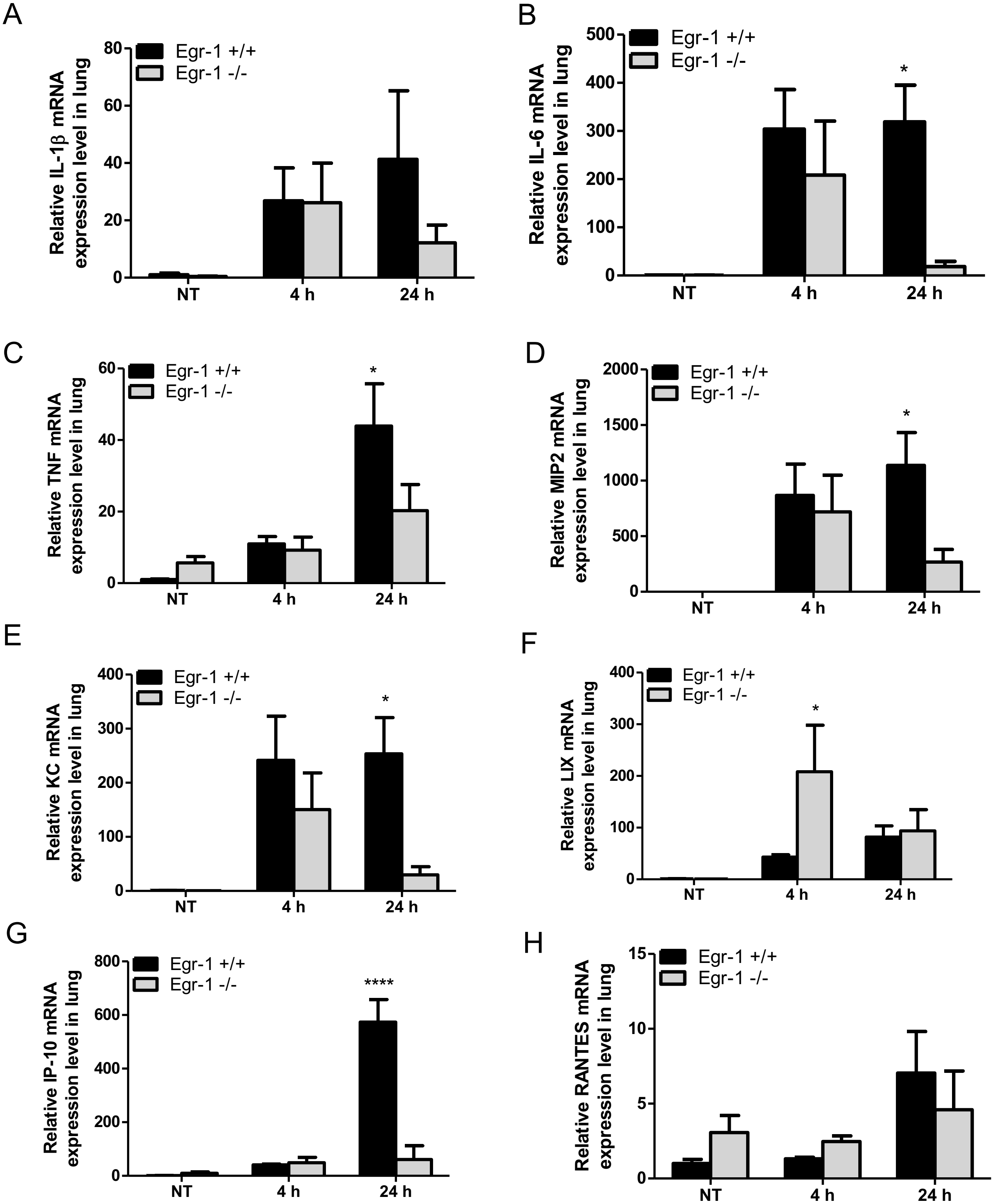

Fig S1. Egr-1 deficiency impairs proinflammatory cytokine mRNA transcription but has differential effects on chemokine mRNA transcription during *P. aeruginosa* lung infection. Wild-type (+/+) and Egr-1-deficient (-/-) mice were intranasally infected with 1 X 10^9 CFU/mouse of *P. aeruginosa* 8821 for 4 h, 24 h or an equivalent volume of saline as a control (NT). The total RNA extracted from lungs was reverse transcribed to cDNA and subjected to real-time quantitative PCR for *IL-1β* (A), *IL-6* (B), *TNF* (C), *MIP2* (D), *KC* (E), *LIX* (F), *IP-10* (G) and *RANTES* (H) gene expression. The gene expression was normalized to housekeeping control gene HPRT ($n = 3 \pm$ SEM, $^*p < 0.05$).

Fig S2

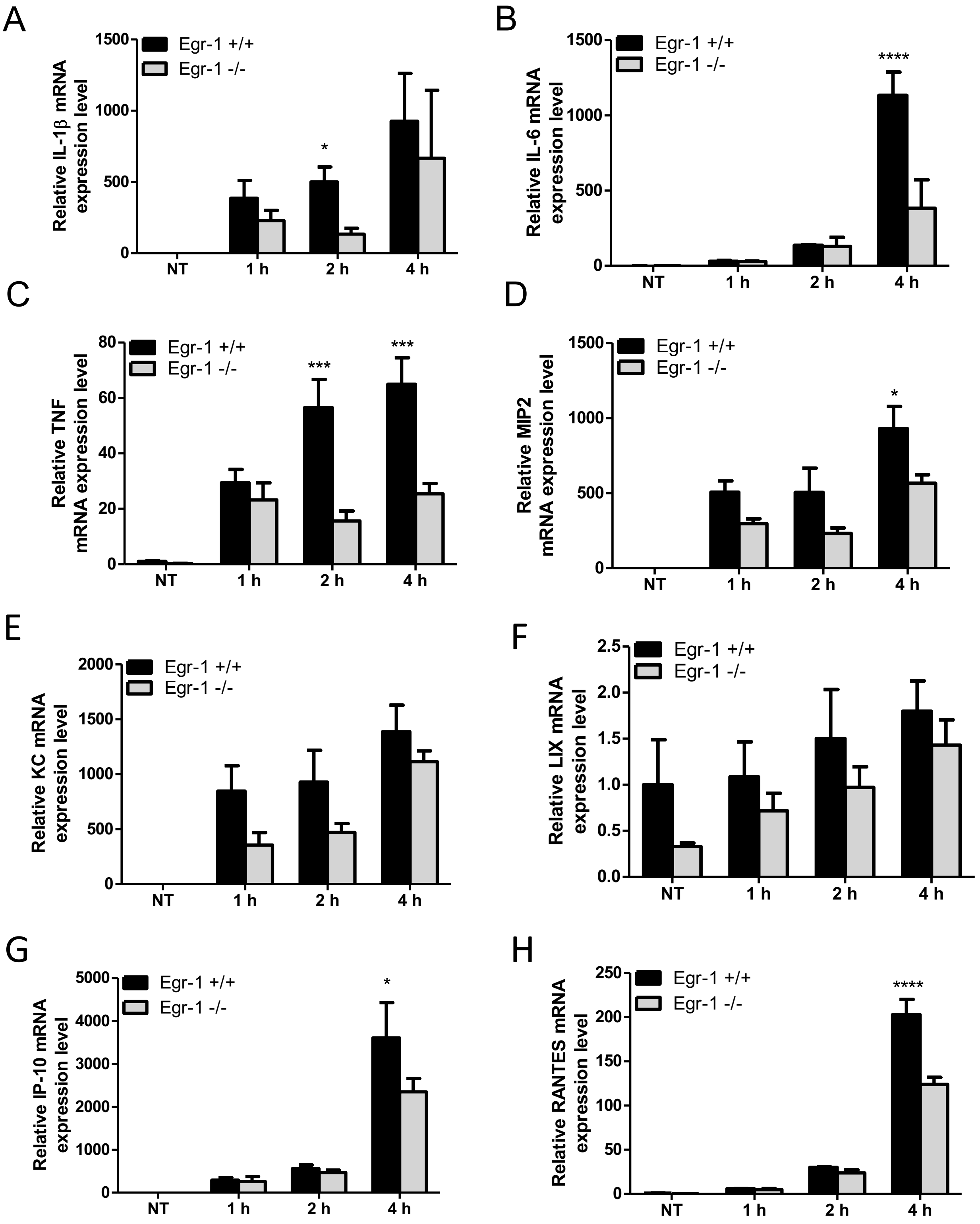

Fig S2. Egr-1-deficient BMMs display impaired proinflammatory cytokine and chemokine production following *P. aeruginosa* infection. Wild-type (+/+) and Egr-1-deficent (-/-) BMMs were infected with *P. aeruginosa* 8821 at a MOI of 10 for 1 h, 2 h, 4 h or left untreated (NT). Total RNA isolated from these cells was reverse transcribed to cDNA and subjected to real-time quantitative PCR for *IL-1β* (A), *IL-6* (B), *TNF* (C), *MIP2* (D), *KC* (E), *LIX* (F), IP-10 (G) and RANTES (H) gene expression. The gene expression was normalized to housekeeping control gene HPRT (n = 3 ± SEM, *p < 0.05, ***p < 0.001, ****p < 0.0001).

Fig S3

PAO1-infected Macrophages

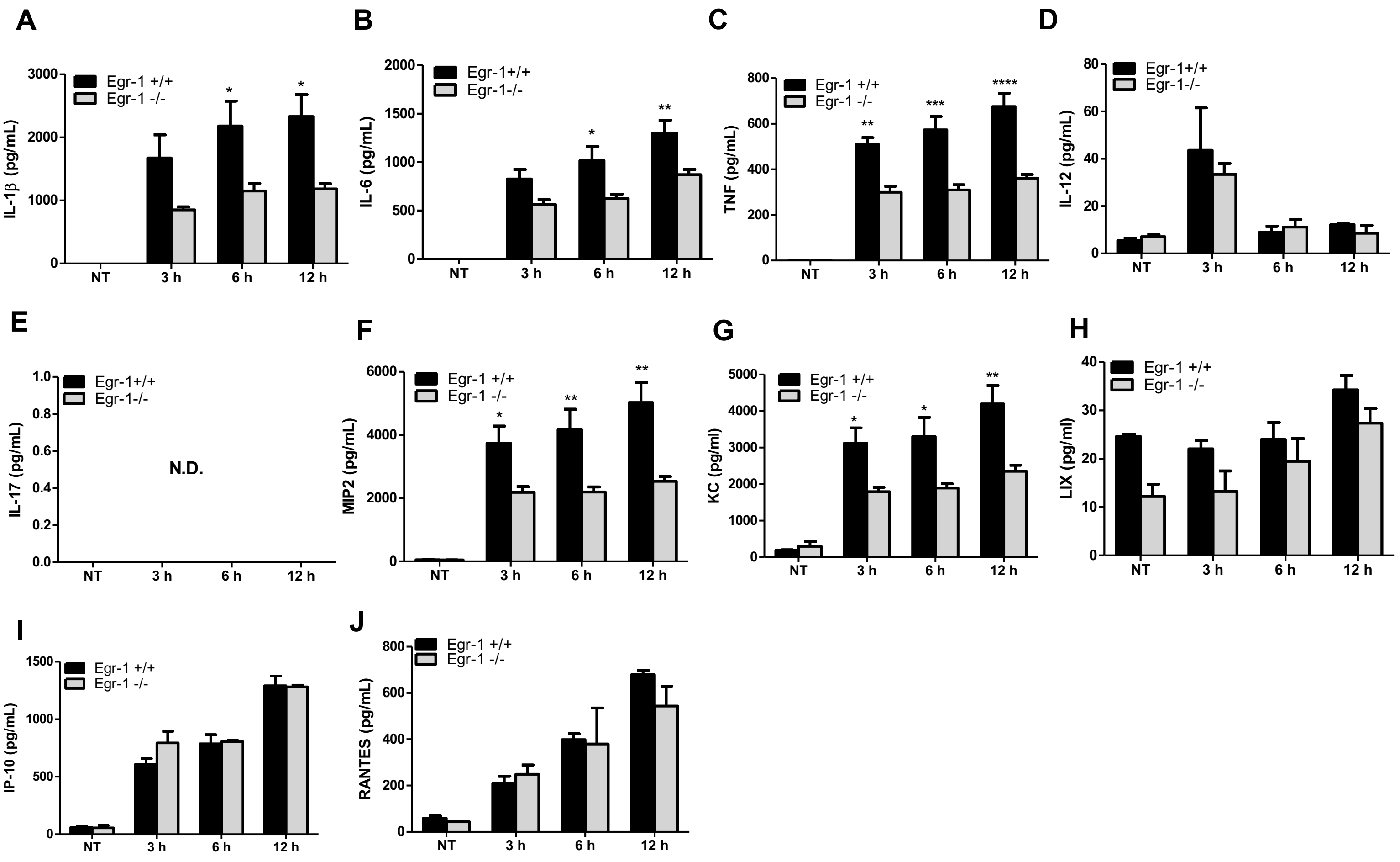

Fig S3. Egr-1-deficient BMMs display impaired proinflammatory cytokine and chemokine production following *P. aeruginosa* PAO1 infection. Wild-type (+/+) and Egr-1-deficient (-/-) BMMs were infected with *P. aeruginosa* PAO1 at a MOI of 10 for 3 h, 6 h, 12 h or left untreated (NT). Cell supernatants were collected for the determination of IL-1β (A), IL-6 (B), TNF (C), IL-12 (D), IL-17 (E), MIP2 (F), KC (G), LIX (H), IP-10 (I) and RANTES (J) secretion by ELISA (n = 3 ± SEM, *p < 0.05, **p < 0.01, ***p < 0.001, N.D. = not detected).

Fig S4

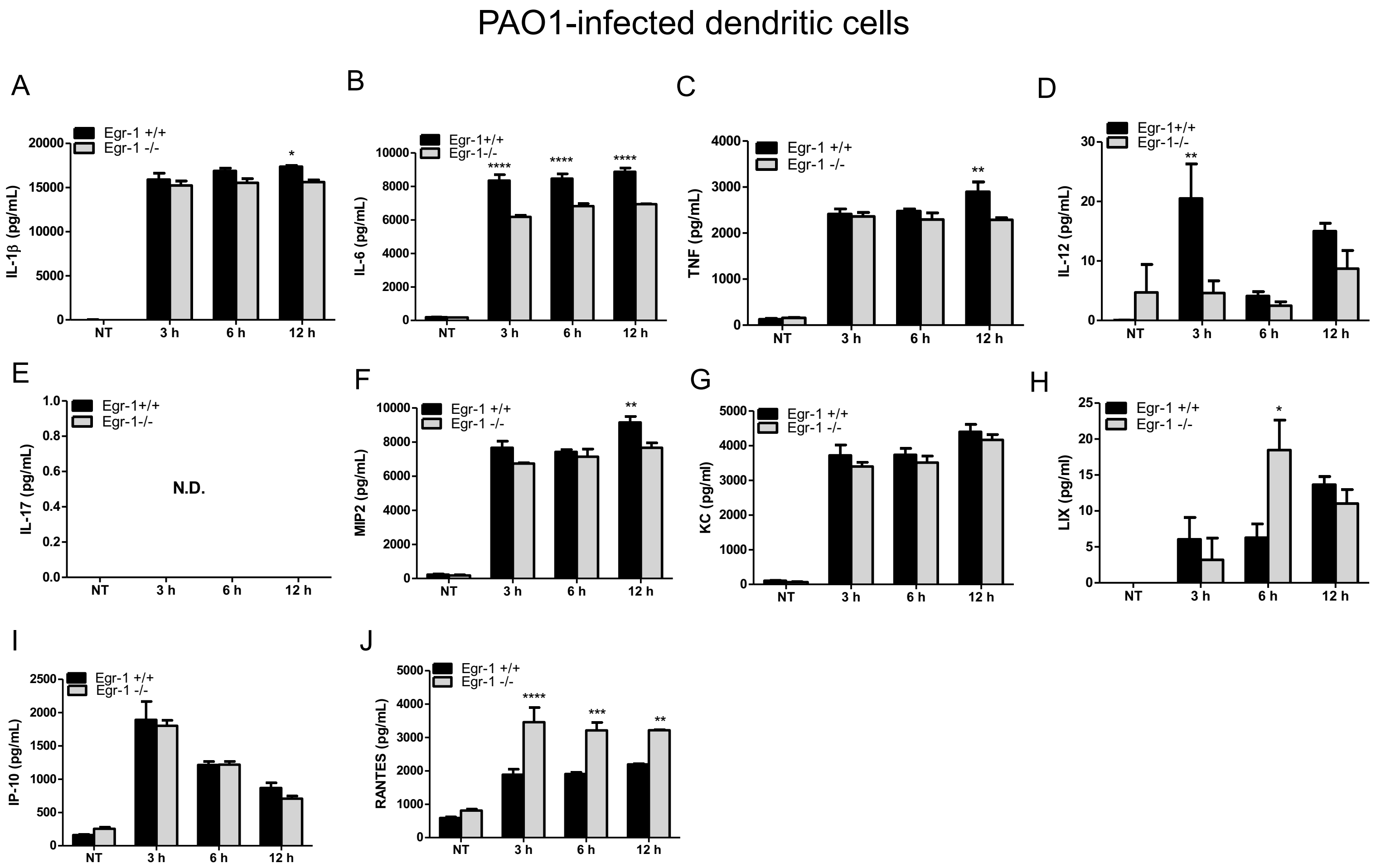

Fig S4. Egr-1-deficient BMDCs have increased LIX production following *P. aeruginosa* PAO1 infection. Wild-type (+/+) and Egr-1-deficient (-/-) BMDCs were infected with *P. aeruginosa* PAO1 at a MOI of 10 for 3 h, 6 h, 12 h or left untreated (NT). Cell supernatants were collected for the determination of IL-1β (A), IL-6 (B), TNF (C), IL-12 (D), IL-17 (E), MIP2 (F), KC (G), LIX (H), IP-10 (I) and RANTES (J) secretion by ELISA. ($n = 3 \pm$ SEM, *$p < 0.05$, **$p < 0.01$, ***$p < 0.001$, ****$p < 0.0001$, N.D. = not detected).

Fig S5

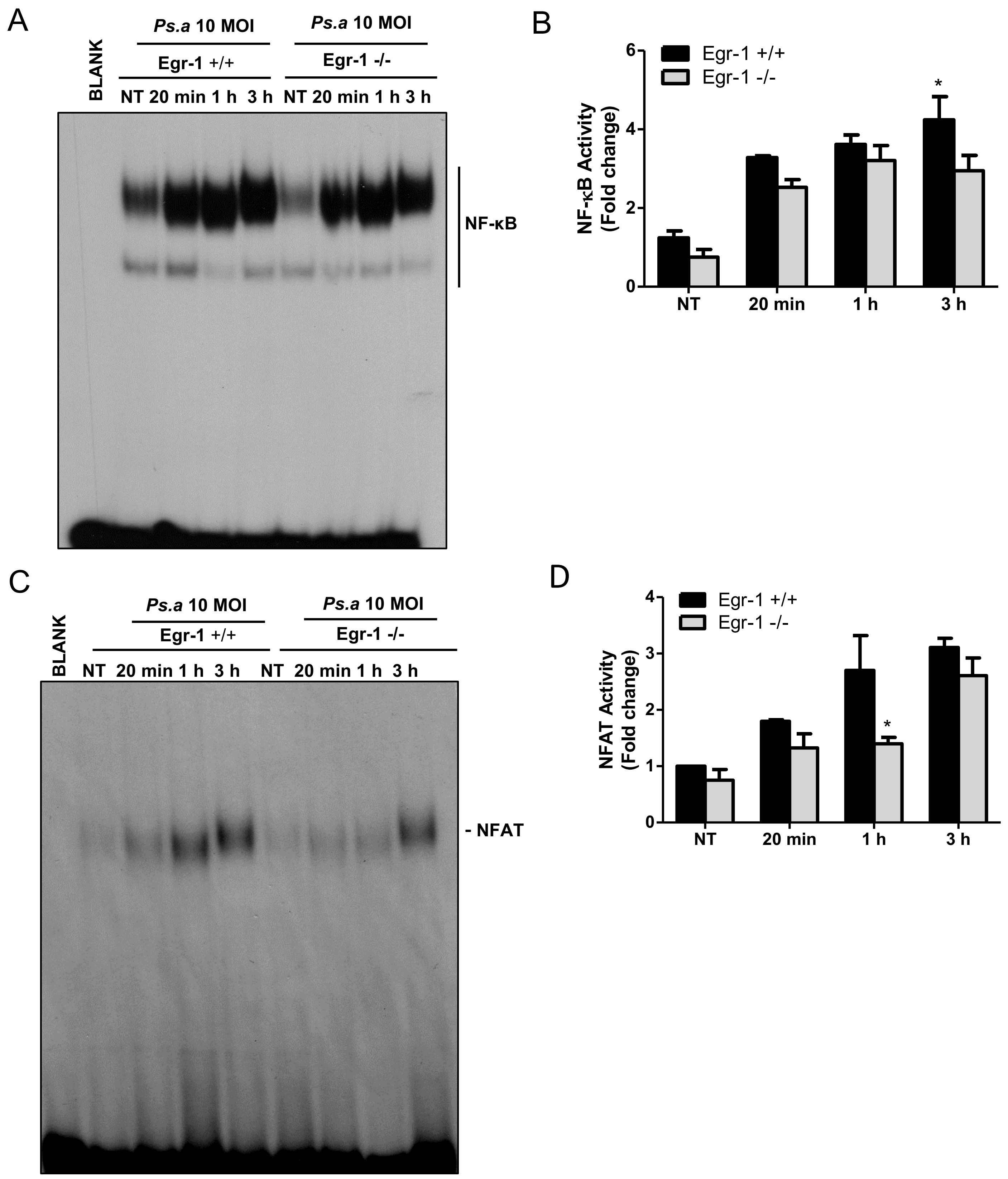

Fig S5. Egr-1-deficient BMMs display diminished NF-κB and NFAT activation during *P. aeruginosa* infection. Wild-type (+/+) and Egr-1-deficient (-/-) BMMs were infected with *P. aeruginosa* 8821 at a MOI of 10 for 20 min, 1 h, 3 h or left untreated (NT). Nuclear proteins were extracted and subjected to EMSA by incubation with ^{32}P-labeled NF-κB (A) and NFAT (C) DNA probes. Data are representative of three individual experiments. Densitometry analysis was performed for NF-κB (B) and NFAT (D) activities, and data are expressed as fold change data versus wild-type untreated BMMs (n = ± 3 SEM, *$p < 0.05$).

Fig S6

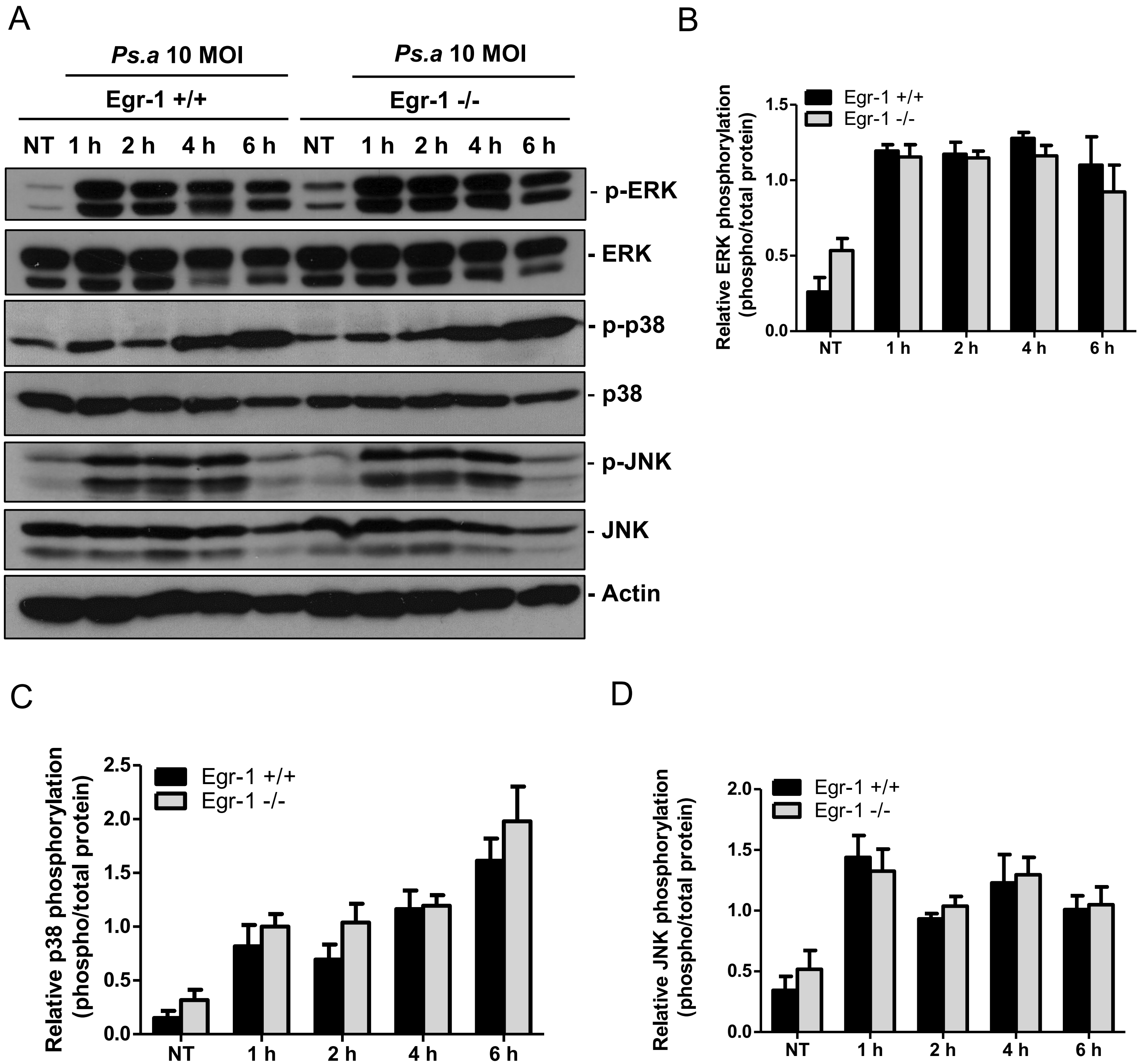

Fig S6. Egr-1 deficiency has no effect on MAPK activation in macrophages during *P. aeruginosa* infection. Wild-type (+/+) and Egr-1-deficient (-/-) BMMs were infected with *P. aeruginosa* 8821 at a MOI of 10 for 1 h, 2 h, 4 h, 6 h or left untreated (NT). Cell lysates were subjected to Western blot analysis for determining phospho- and total ERK, p38 and JNK, as well as actin as a loading control. Blots are representative of three independent experiments (A). Densitometry analysis of phosphorylated ERK (B), p38 (C) and JNK (D) was normalized to their total protein respectively (n = 3 ± SEM).

Fig S7

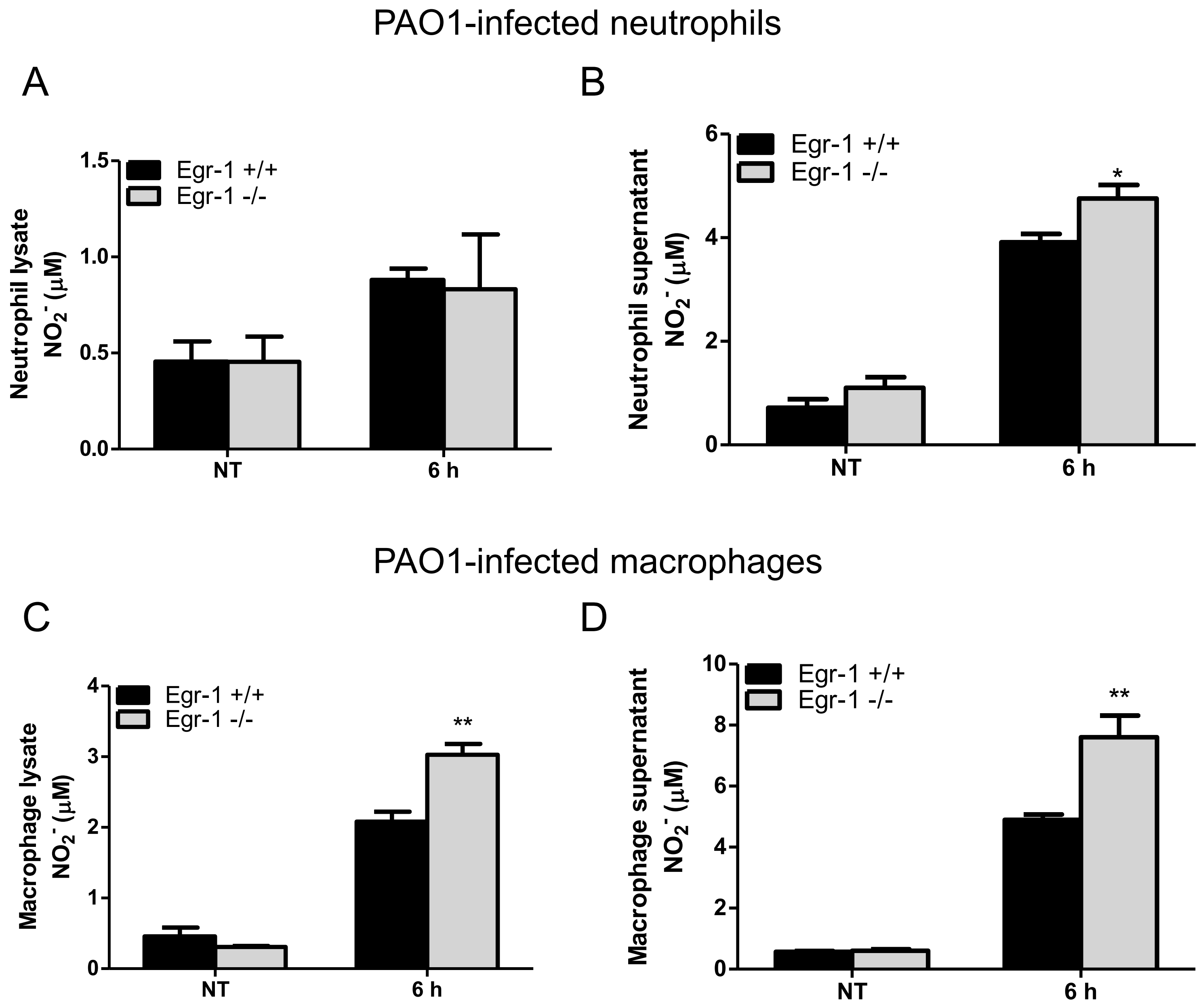

Fig S7. Egr-1 deficiency leads to increased nitric oxide production in neutrophils and macrophages in response to *P. aeruginosa* PAO1 infection. Wild-type (+/+) and Egr-1-deficient (-/-) neutrophils and BMMs were infected with *P. aeruginosa* PAO1 for 6 h or or left untreated (NT). The NO_2^- levels were tested in cell lysates (A, C) and supernatants (B, D) at 6 h (n = 3 ± SEM, *$p < 0.05$, **$p < 0.01$).

Fig S8

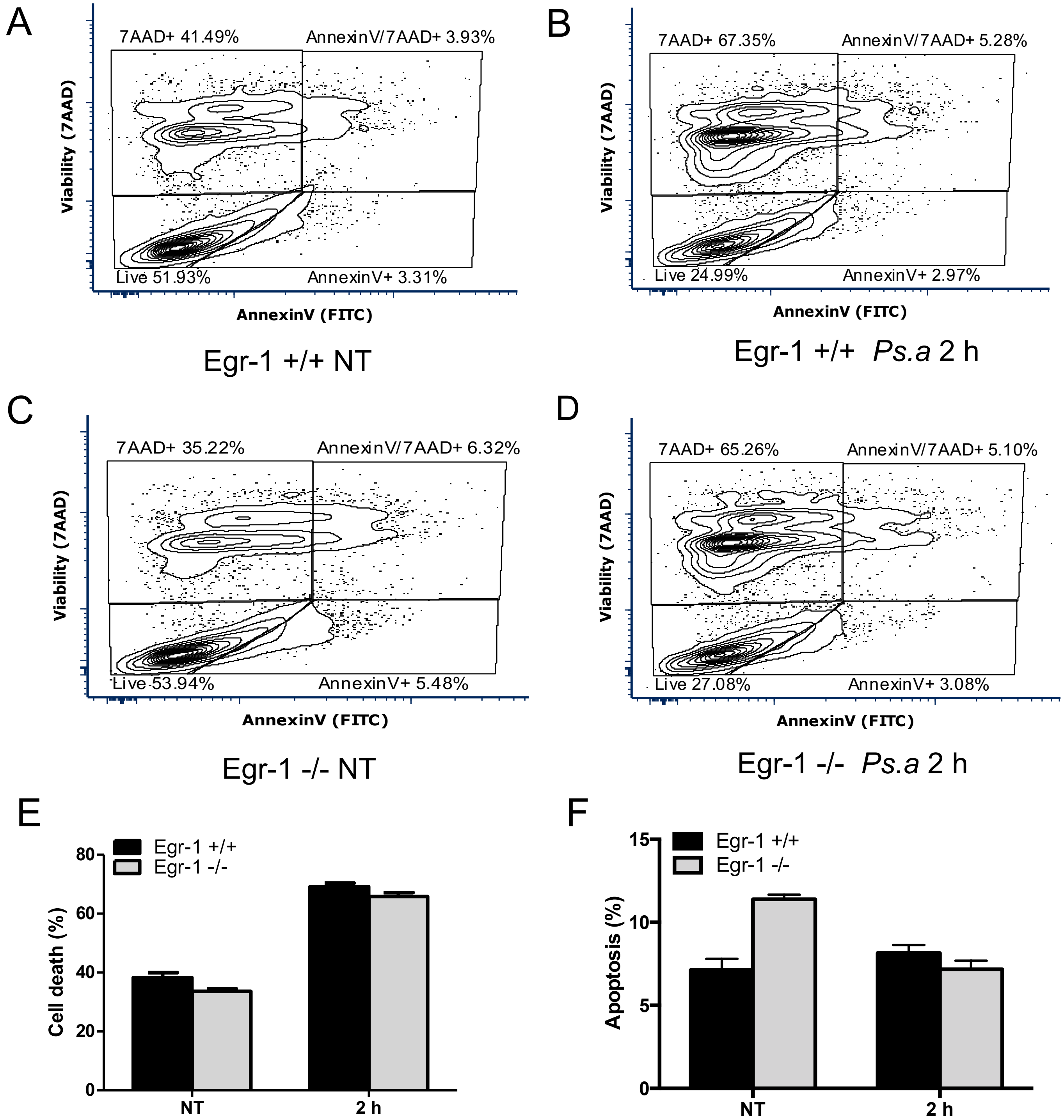

Fig S8. Egr-1 deficiency has no impact on macrophage apoptosis during *P. aeruginosa* infection. Wild-type (+/+) and Egr-1-deficient (-/-) BMMs were infected with *P. aeruginosa* 8821 at a MOI of 10 for 2 h or left untreated (NT). The cells were stained with FITC Annexin V and 7-AAD, and a total 2 X 10^4 cells from each sample was analyzed on a flow cytometer. The density plots for wild-type (A and B) and Egr-1-deficient (C and D) macrophages were represented of three individual experiments. The bar graphs for the data of cell death (E) and apoptosis (F) were presented as percentage (n = 3).
